# Three human RNA polymerases interact with TFIIH via a common RPB6 subunit

**DOI:** 10.1093/nar/gkab612

**Published:** 2021-07-16

**Authors:** Masahiko Okuda, Tetsufumi Suwa, Hidefumi Suzuki, Yuki Yamaguchi, Yoshifumi Nishimura

**Affiliations:** Graduate School of Medical Life Science, Yokohama City University, 1-7-29 Suehiro-cho, Tsurumi-ku, Yokohama 230-0045, Japan; School of Life Science and Technology, Tokyo Institute of Technology, Yokohama, 226-8501, Japan; School of Life Science and Technology, Tokyo Institute of Technology, Yokohama, 226-8501, Japan; School of Life Science and Technology, Tokyo Institute of Technology, Yokohama, 226-8501, Japan; Graduate School of Medical Life Science, Yokohama City University, 1-7-29 Suehiro-cho, Tsurumi-ku, Yokohama 230-0045, Japan; Graduate School of Integrated Sciences for Life, Hiroshima University, 1-4-4 Kagamiyama, Higashi-Hiroshima 739-8258, Japan

## Abstract

In eukaryotes, three RNA polymerases (RNAPs) play essential roles in the synthesis of various types of RNA: namely, RNAPI for rRNA; RNAPII for mRNA and most snRNAs; and RNAPIII for tRNA and other small RNAs. All three RNAPs possess a short flexible tail derived from their common subunit RPB6. However, the function of this shared N-terminal tail (NTT) is not clear. Here we show that NTT interacts with the PH domain (PH-D) of the p62 subunit of the general transcription/repair factor TFIIH, and present the structures of RPB6 unbound and bound to PH-D by nuclear magnetic resonance (NMR). Using available cryo-EM structures, we modelled the activated elongation complex of RNAPII bound to TFIIH. We also provide evidence that the recruitment of TFIIH to transcription sites through the p62–RPB6 interaction is a common mechanism for transcription-coupled nucleotide excision repair (TC-NER) of RNAPI- and RNAPII-transcribed genes. Moreover, point mutations in the RPB6 NTT cause a significant reduction in transcription of RNAPI-, RNAPII- and RNAPIII-transcribed genes. These and other results show that the p62–RPB6 interaction plays multiple roles in transcription, TC-NER, and cell proliferation, suggesting that TFIIH is engaged in all RNAP systems.

## INTRODUCTION

In eukaryotic transcription, three RNAPs are essential for synthesizing various RNAs: RNAPI for ribosomal RNA (rRNA), RNAPII for messenger RNA (mRNA) and most small nuclear RNAs (snRNAs), and RNAPIII for transfer RNA (tRNA) and other small RNAs. The three RNAPs are multi-subunit complexes comprising five common subunits and several specific subunits, in total more than twelve subunits. Among the latter subunits, RPB1 is specific to RNAPII and has a well-characterized long flexible C-terminal domain (CTD) tail that provides a critical binding platform for various transcription factors and RNA processing factors (1). CTD comprises heptad repeats of a sequence ‘YSPTSPS’, which is repeated 52 times in humans and 26 times in budding yeast (2), and its phosphorylation is important for regulating multiple stages in transcription (1).

The general transcription factor TFIIH is a multifunctional 10-subunit complex involved in transcription, NER and cell cycle (3). The XPB and XPD subunits possess the ATPase/helicase activities necessary for opening promoter DNA during transcription initiation and damaged DNA during NER. The CDK7 subunit phosphorylates S5 of the YSPTSPS consensus repeats of RNAPII CTD, other transcription factors, and nuclear receptors. Moreover, it has been shown that the p62 subunit plays a crucial role in recruiting TFIIH to appropriate functional sites through the N-terminal pleckstrin homology (PH) domain (PH-D) interacting with intrinsically disordered acidic regions of various transcription factors such as TFIIEα (4,5), p53 (6,7), EKLF (8), DP1 (9), VP16 (10,11) and EBNA2 (12) and several NER factors such as XPC (13,14), UVSSA (15) and XPG (16,17).

Here, we show that p62 PH-D also interacts with a short tail of RNAP, derived from the N-terminal tail (NTT) of RPB6, a subunit shared by all three RNAPs. We determined the solution structures of RPB6, both in a free form and when bound to p62 PH-D by NMR spectroscopy. On the basis of the NMR structures and previously solved cryo-electron microscopy (cryo-EM) structures (18–20), we built reasonable structural models of RNAPII bound to TFIIH and also the activated elongation complex of RNAPII bound to TFIIH. Point mutations in the RPB6 NTT that impair p62 binding resulted in cell growth defects associated with significant reduction in transcription of RNAPI-, RNAPII- and RNAPIII-transcribed genes and defects in TC-NER of RNAPI- and RNAPII-transcribed genes, indicating that TFIIH is engaged in all RNAP systems through the RPB6–TFIIH p62 interaction.

## MATERIALS AND METHODS

### Preparation of human p62 PH domain (PH-D)

Unlabeled or ^13^C/^15^N-labeled human TFIIH p62 PH-D (residues 1–108) and unlabeled or ^13^C/^15^N-labeled human RPB6 (residues 1–127) were prepared as previously described (5). In brief, p62 or RPB6 was expressed as a hexa-histidine-tagged product in a pET15b vector (Merck Millipore) in *Escherichia coli* BL21 (DE3) Gold (Agilent Technologies). The lysed supernatant was loaded onto a Ni-nitrilotriacetic acid (NTA) column (QIAGEN), and the eluate was digested with thrombin to remove the histidine tag. After concentration with an Amicon Ultra device (Merck Millipore), the sample was purified on a Superdex75 column (GE Healthcare).

### Isothermal titration calorimetry

The binding dissociation constant (*K*_d_) for the interaction between p62 PH-D and human RPB6 (residues 1−24) or yeast (*Saccharomyces cerevisiae*) Rpb6 (residues 11−34) was measured by ITC using a VP-ITC calorimeter (MicroCal). Titration of 100∼300 μM RPB6/Rpb6 in the syringe (25 × 20 μl injections) into 2 ml of 10–30 μM p62 PH-D in the cell was carried out in 20 mM potassium phosphate (pH 6.8) with or without 25 mM NaCl at 20°C. Each injection took 4 s, with a pre-injection delay of 210 s and a syringe stirring speed of 307 rpm. Data were analyzed by using the Origin software package (MicroCal).

### NMR titration

RPB6 (residues 1−127) dissolved in 20 mM potassium phosphate (pH 6.8), 25 mM NaCl, 5 mM deuterated DTT and 10% D_2_O was added to 50 μM ^15^N-labeled p62 PH-D at the following molar ratios: 1:0, 1:0.25, 1:0.50, 1:0.75, 1:1.00, 1:1.50, 1:2.00 and 1:2.50. ^1^H,^15^N-HSQC spectra were acquired before and after addition of RPB6 at 32°C on an AVANCE III HD 600-MHz spectrometer (Bruker) equipped with a Cryo-TCI probe. The chemical shift change Δδ was plotted as a function of molar ratio. *K*_d_ was calculated by employing the nonlinear regression fitting function:

$$\begin{equation*}\Delta {\rm{\delta = \Delta }}{{\rm{\delta }}_{{\rm{max}}}}\left( {{K_{\rm{d}}} + {\rm{ }}{{\left[ {\rm{P}} \right]}_{\rm{t}}}{\rm{ + }}{{\left[ {\rm{L}} \right]}_{\rm{t}}}-{\rm{ }}{{\{ {{({K_{\rm{d}}} + {\rm{ }}{{\left[ {\rm{P}} \right]}_{\rm{t}}}{\rm{ + }}{{\left[ {\rm{L}} \right]}_{\rm{t}}}{\rm{)}}}^2}-{\rm{ }}\left( {{\rm{4}}{{\left[ {\rm{P}} \right]}_{\rm{t}}}{{\left[ {\rm{L}} \right]}_{\rm{t}}}} \right)\} }^{1/2}}} \right){\rm{/2}}{\left[ {\rm{P}} \right]_{\rm{t}}}\end{equation*}$$

where Δδ_max_ is the maximal change in chemical shift, and [P]_t_ and [L]_t_ are the total concentrations of protein and ligand, respectively. In this study, [P]_t_ and [L]_t_ corresponded to, respectively, ^15^N-labeled p62 PH-D and unlabeled RPB6. Signals from Glu58, Gln66, Thr74, Phe77 and Lys102 of p62 PH-D were used to calculate *K*_d_.

### NMR structure determination

To determine the structure of RPB6, we used 2 mM ^13^C/^15^N-labeled RPB6 in 20 mM potassium phosphate (pH 6.8), 25 mM NaCl, and 5 mM deuterated DTT, prepared in either 90% H_2_O/10% D_2_O or 99.9% D_2_O. NMR experiments were performed at 25°C on AVANCE III HD 600-MHz and 950-MHz spectrometers (Bruker), each equipped with a Cryo-TCI probe. Backbone and side-chain resonances were assigned by using standard triple-resonance NMR experiments (21). Stereospecific assignments were obtained from a combination of HNHB, HN(CO)HB, HNCG, HN(CO)CG and ^13^C-edited and ^15^N-edited NOESY-HSQC spectra. Distance restraints were obtained from ^15^N-edited NOESY-HSQC (τ_m_ = 150 ms) and ^13^C-edited NOESY-HSQC (τ_m_ = 50 and 100 ms) spectra. Side-chain torsion angles, χ1 and χ2, were obtained from a combination of HNHB, HN(CO)HB, HNCG, HN(CO)CG, and ^13^C-edited and ^15^N-edited NOESY-HSQC spectra. Hydrogen bond restraints were obtained by backbone amide H/D-exchange experiments. Spectra were processed by using NMRPipe (22) and analyzed by using NMRView (23).

To determine the structure of RPB6 bound to p62 PH-D, the complex was prepared at 0.38 mM by mixing ^13^C/^15^N-labeled RPB6 with unlabeled p62 PH-D at a molar ratio of 1.0:2.0 in 20 mM potassium phosphate (pH 6.8), 25 mM NaCl, 5 mM deuterated DTT, and either 10% D_2_O or 99.9% D_2_O. In the same way, ^13^C/^15^N-labeled p62 PH-D was mixed with unlabeled RPB6. NMR experiments were performed at 25°C on AVANCE III HD 600-MHz and 950-MHz spectrometers (Bruker), each equipped with a Cryo-TCI probe. Backbone and side-chain resonances were assigned by using standard triple-resonance NMR experiments (21). Stereospecific assignments were obtained from a combination of HNHB, HN(CO)HB, HNCG, HN(CO)CG and ^13^C-edited and ^15^N-edited NOESY-HSQC spectra. Intramolecular distance restraints were obtained from ^15^N-edited NOESY-HSQC (τ_m_ = 50 and 150 ms) and ^13^C-edited NOESY-HSQC (τ_m_ = 50 and 100 ms) spectra. Intermolecular distance restraints were obtained from ^13^C,^15^N-filtered/edited NOESY (τ_m_ = 120 and 150 ms) spectra. Side-chain torsion angles, χ1 and χ2, were obtained from a combination of HNHB, HN(CO)HB, HNCG, HN(CO)CG, and ^13^C-edited and ^15^N-edited NOESY-HSQC (τ_m_ = 50 ms) spectra. Hydrogen bond restraints were obtained by backbone amide H/D-exchange experiments. Spectra were processed by using NMRPipe (22) and analyzed by using NMRView (23).

### Structure calculation

Interproton distance restraints derived from NOE intensities were grouped into four distance ranges: 1.8−2.7 Å (1.8−2.9 Å for NOEs involving HN protons), 1.8−3.3 Å (1.8−3.5 Å for NOEs involving HN protons), 1.8−5.0 and 1.8−6.0 Å, corresponding to strong, medium, weak, and very weak NOEs, respectively. The upper limit was corrected for constraints involving methyl groups, aromatic ring protons, and non-stereospecifically assigned methylene protons. Dihedral angle restraints for φ and ψ were obtained from analysis of the backbone chemical shifts with TALOS+ (24). χ1 and χ2 angles were restrained ±30° for three side-chain rotamers. Structure calculations were performed by distance geometry and simulated annealing using the program Xplor-NIH (25,26). In total, we calculated 100 structures, which were each subjected to water refinement (27) by immersion in a 7.0-Å layer of water molecules. After minimization with 120 steps, we carried out a heating stage from 100 to 500 K with 200 steps of molecular dynamics for every 100-K increment, a refinement stage with 2500 steps at 500 K, and a cooling stage from 500 to 25 K with 200 steps for every 25-K decrement. The refinement protocol was finished with 200 steps of minimization. Statistics for the 20 best structures are summarized in Table 1. Structures were analyzed and displayed by using PROCHECK-NMR (28), MOLMOL (29) and PyMol (http://www.pymol.org).

**Table 1.** Statistics for the 20 best structures of RPB6 and the complex formed between RPB6 and TFIIH p62 PH-D

	Free	Complex		
	RPB6	RPB6	TFIIH p62 PH-D	
Experimental restraints				
Total NOE	1783	1584	2188	
Intraresidue	279	279	365	
Sequential (*i* – *j* = 1)	522	460	501	
Medium-range (1 < *i* – *j* < 5)	361	326	365	
Intramolecular long-range (*i* – *j* ≥ 5)	621	519	957	
Intermolecular		174		
Hydrogen bond	35×2	27×2	46×2	
Number of dihedral restraints				
φ	110	109	97	
ψ	110	104	96	
χ1	47	47	60	
χ2	7	7	10	
Statistics for structure calculations				
R.m.s. deviations from experimental restraints^a^				
Distance (Å)	0.037 ± 0.001	0.039 ± 0.001		
Dihedral (°)	0.337 ± 0.063	0.442 ± 0.049		
R.m.s. deviations from idealized covalent geometry				
Bonds (Å)	0.00472 ± 0.00008	0.00523 ± 0.00010		
Angles (°)	0.579 ± 0.015	0.663 ± 0.011		
Improper (°)	0.588 ± 0.018	0.705 ± 0.020		
Coordinate precision Average pairwise r.m.s. deviation from the mean structure				
Backbone atoms (Å)	0.53 ± 0.15^b^	0.61 ± 0.14^c^	0.51 ±0.10^d^	0.39 ±0.07^e^
Heavy atoms (Å)	1.09 ±0.18^b^	1.11 ± 0.17^c^	1.14 ±0.18^d^	1.01 ±0.17^e^
Ramachandran plot statistics				
Residues in most favored regions (%)	83.4^f^	81.6^g^		
Residues in additional allowed regions (%)	16.0^f^	17.6^g^		
Residues in generously allowed regions (%)	0.1^f^	0.6^g^		
Residues in disallowed regions (%)	0.5^f^	0.2^g^		

^a^None of the structures exhibited distance violations >0.5 Å, dihedral angle violations >5°.

^b^The value was calculated over residues 36–39 and 53–125 of the RPB6.

^c^The value was calculated over residues 36–39 and 53–125 of the RPB6 in the complex.

^d^The value was calculated over residues 3–19 of the RPB6 and residues 7–104 of the TFIIH p62 PH-D in the complex.

^e^The value was calculated over residues 7–104 of the TFIIH p62 PH-D in the complex.

^f^The value was calculated over residues 36–125 of the RPB6.

^g^The value was calculated over residues 3–125 of the RPB6 and residues 7–104 of the TFIIH p62 PH-D in the complex.

### NMR relaxation analysis

The steady-state ^15^N–{^1^H} NOE values were measured by using ^13^C/^15^N-labeled RPB6 (2.0 mM) at 25°C on an AVANCE III HD a 600-MHz spectrometer (Bruker) equipped with a Cryo-TCI probe, and were determined from peak intensity ratios obtained from spectra acquired with and without proton saturation. Uncertainties were determined from the standard deviation in background noise levels by using NMRView (23).

### Structural docking modeling

To build a structural model of RNAPII docking with p62 PH-D or TFIIH, the N-terminal 41 residues of human RPB6 from our NMR structure (PDB code 7DTI) were linked to RPB6 of human RNAPII from the Cryo-EM structure (PDB code 5IY6) at Gly42 by using the program PyMol (http://www.pymol.org). To build a structural model of the RNAPII–elongation complex docking with TFIIH, the N-terminal 45 residues of human RPB6 from our NMR structure (PDB code 7DTI) were linked to RPB6 of pig RNAPII from the Cryo-EM structure (PDB code 6GMH) at Gln46 by using PyMol. For the human TFIIH Core complex, we used a previously reported model (30). Docking calculations were done by the program Xplor-NIH (25,26) using the scripts ‘rigid_min.inp’ and ‘sa_cross_tor.inp’. We employed the experimentally obtained intermolecular distance restraints used in the structure determination of the RPB6–p62 PH-D complex.

### Cell culture

HeLa cells were cultured in DMEM supplemented with 10% FBS and antibiotics at 37°C at 5% CO_2_. To knock down RPB6, three synthetic double-stranded oligonucleotides encoding shRNAs against RPB6 (listed in Supplementary Table S1) were inserted into the pRSI9 control vector, which was derived from pRSI9-U6-(sh)-UbiC-TagRFP-2A-Puro (Cellecta). To express exogenous untagged WT or mutant RPB6, first RNAi-resistant RPB6 was prepared by gene synthesis, subcloned into pLenti6 (Thermo Fisher Scientific), and used as a template to introduce amino acid substitutions by inverse PCR. Recombinant viral particles were then produced in 293FT cells using the resultant plasmids and ViraPower Lentiviral Packaging Mix (Thermo Fisher Scientific). HeLa cells were infected with the appropriate RPB6 expression vector (WT, F13A, F8A-F13A or ΔN20) and cultured in the presence of 3 μg/ml of blasticidin (InvivoGen) for 1 week. The cells were then further infected with one of shRPB6 expression vectors, and stably transduced cells were selected by incubation with 0.5 μg/ml of puromycin for another week (Thermo Fisher Scientific). The most effective shRNA (shRPB6 #2) was used in most experiments. In some experiments, the resultant cells were treated with the RNAP inhibitor BMH21 (AdooQ Bioscience) or ML60218 (FOCUS Biomolecules) dissolved in DMSO.

### RNA extraction and qRT-PCR analysis

Total RNA was extracted by using Sepasol-RNA I Super G (Nacalai Tesque). qRT-PCR reactions were carried out by using a One Step TB Green PrimeScript RT-PCR Kit (Takara), the appropriate primer set (Supplementary Table S1), and a StepOnePlus Real Time PCR System (Thermo Fisher Scientific). Unlike the standard procedure based on a reference gene, total RNA samples prepared from the same number of cells were subjected to qRT-PCR and compared directly, because there were no genes that were not affected by RPB6 knockdown.

### RNA-seq

RNA-seq was performed in triplicate. Total RNA was extracted by using Sepasol-RNA I Super G and further purified using an RNeasy Mini Kit (Qiagen). Libraries were prepared by using a SureSelect Strand Specific RNA Library Preparation Kit (Agilent), and 75-cycle single-read sequencing was carried out with a NextSeq 500 (Illumina) and NextSeq 500/550 High Output Kit v2.5 (Illumina). The obtained reads (>10,000,000 per sample) were analyzed by using CLC Genomics Workbench version 11.0 (Qiagen). The reads were mapped to the human genome assembly GRCh38, and TPMs were used to identify differentially expressed genes with a minimum fold change of 2 and a maximum FDR *q*-value of 0.05. Gene set enrichment analysis was performed with the Functional Annotation Tool DAVID.

### Measurement of cell viability after UV irradiation

HeLa cells cultured on 35-mm dishes were washed with PBS and irradiated with 254 nm UV-C light using a Stratalinker 2400 (Stratagene). After further culture at 37°C for 72 h, the numbers of viable cells were counted by using a hemocytometer.

### Measurement of cell viability after cisplatin treatment

HeLa cells cultured on 96-well plates were treated with various concentrations of cisplatin (Tokyo Chemical Industry) for 72 h. Next, 10 μl of Cell Count Reagent SF (Nacalai Tesque) was added to each well, and absorbance at 450 and 600 nm was measured with a GloMax-Multi Detection System (Promega) after 1 h of incubation at 37°C.

### Fluorescence-based assays for RRS and UDS

Fluorescence-based RRS and UDS assays were performed essentially as described (31). HeLa cells grown on coverslips were treated with 100 μM cisplatin for 2 h or irradiated with UV-C light as described above. After 0, 6 or 24 h of incubation in complete medium, the damaged cells were further cultured in serum-free DMEM containing 100 μM 5-ethynyluridine (Toronto Research Chemicals) for 1 h (for RRS) or in serum-free DMEM containing 10 μM 5-ethynyl-2′-deoxyuridine (Tokyo Chemical Industry) for 2 h (for UDS). After fixation and permeabilization with 2% paraformaldehyde and 0.5% Triton X-100, the resultant cells were incubated with 25 μM Alexa Fluor 488-azide (Thermo Fisher Scientific), 4 mM CuSO_4_, and 10 mM sodium ascorbate for 1 h at room temperature in the dark. The cover slips were then washed and mounted by using Vectashield Mounting Medium with DAPI (Vector Laboratories). Fluorescence images were taken with an LSM780 confocal microscope (Zeiss) and analyzed with CellProfiler (32). DAPI signals were used to define the nuclear regions of individual cells, and Alexa Fluor 488 signals in individual nuclei were quantified. For UDS, S-phase cells were removed before analysis. Background-subtracted, geometric mean fluorescence intensities were determined for >125 cells per condition for RRS and >250 cells per condition for UDS.

### RRS assay for individual genes

HeLa cells were treated with 100 μM cisplatin for 2 h. After 0, 6 or 24 h of incubation in complete medium, the damaged cells were further cultured in serum-free DMEM containing 100 μM 5-ethynyluridine for 1 h. Similarly, Sf9 cells were incubated in Sf-900 II SFM (Thermo Fisher Scientific) containing 100 μM 5-ethynyluridine for 1 h. After the cells were counted and total RNA was extracted by Sepasol-RNA I Super G, a spike of Sf9 RNA was added to each RNA sample prepared from the same number of HeLa cells. Nascent RNA was biotinylated by using a Click-iT Nascent RNA Capture Kit (Thermo Fisher Scientific), purified with ethanol precipitation, immobilized to Dynabeads MyOne Streptavidin T1 magnetic beads (Thermo Fisher Scientific), and reverse-transcribed by using Superscript III Reverse Transcriptase (Thermo Fisher Scientific) with seven gene- specific primers on the beads. qPCR reactions were carried out by using KAPA SYBR FAST qPCR Kit Master Mix (2×), an ABI Prism (KAPA Biosystem), the appropriate primer set (Supplementary Table S1), and a StepOnePlus Real Time PCR System. The ΔΔCt method was employed to normalize the results to *Spodoptera frugiperda GAPDH* as a reference gene.

## RESULTS AND DISCUSSION

### RPB6 NTT shared by all three RNAPs provides a binding platform for the PH-D of TFIIH p62

**Figure 1.**
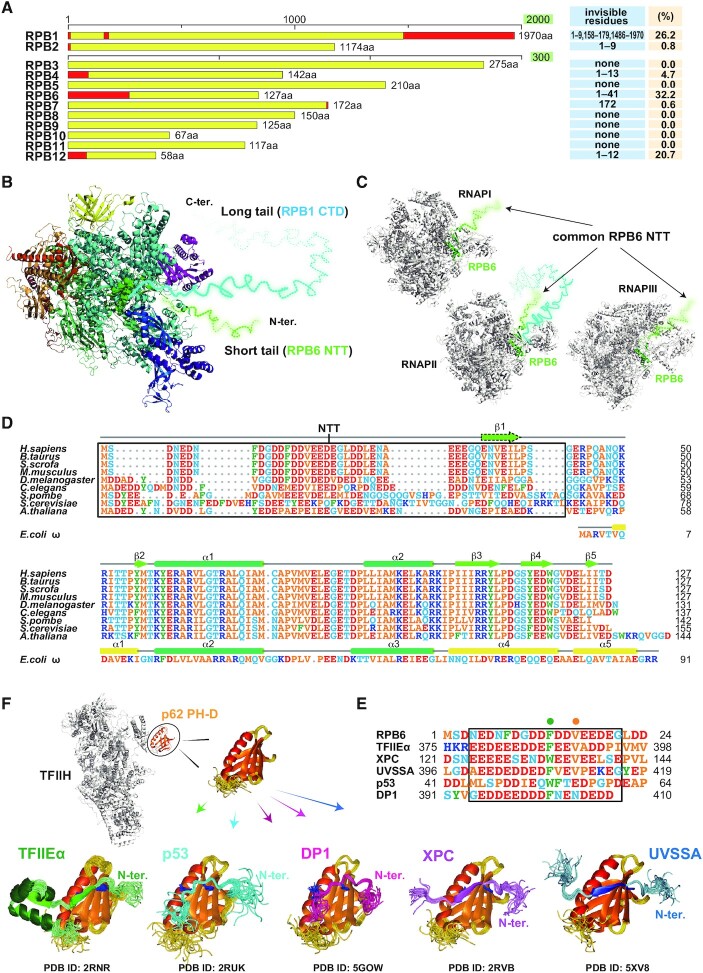
Similarity between the short tail common to RNAPs and the acidic strings of TFIIH p62 PH-D-binding proteins. (**A**) Subunits of human RNAPII. Visible (yellow) and invisible (red) regions in the cryo-EM structure of RNAPII (PDB ID: 5IY6) are indicated. (**B**) Structure of human RNAPII. Invisible RPB1 CTD and RPB6 NTT are drawn freehand as cyan and green broken lines, respectively. (**C**) Structures of yeast RNAPI (PDB ID: 5W65), RNAPII (PDB ID: 5FYW), and RNAPIII (PDB ID: 6EU0). NTT is drawn freehand as a green broken line. (**D**) Amino acid sequence alignment of RPB6 and bacterial (*E. coli*) RNAP subunit ω. NTT residues that are invisible in the cryo-EM structure of human RNAPII (PDB ID: 5IY6) and the corresponding residues in RPB6s of other species are boxed. ω is partially homologous in sequence and structure to RPB6 (43) but lacks NTT. (**E**) Sequence alignment of human RPB6 and p62-binding sites of p62 target proteins. Green and orange dots indicate amino acids required for binding specificity. (**F**) Structures of TFIIH p62 PH-D bound to target proteins.

RNAPII possesses the long flexible CTD tail in RPB1. We noticed that RPB6, a subunit shared by all three RNAPs, similarly has a short flexible tail of unknown function at its N-terminus (NTT) (18,33). To our knowledge, no study has focused on this region. Flexible tails are generally invisible in crystal and/or cryo-EM structures. In the cryo-EM structure of human RNAPII (18), the number of invisible residues is less than 13 in each subunit, except for ∼485 in RPB1 CTD and 41 in RPB6 NTT (Figure 1A and Supplementary Figure S1). Thus, while RNAPII has long and short tails (Figure 1B), RNAPI and RNAPIII have only the short RPB6 NTT (Figure 1C). In the cryo-EM structure of human RNAPII, RPB6 forms a characteristic core, whose amino acid sequence is highly conserved in various species (Figure 1D). Although NTTs are generally divergent, vertebrate NTTs contain highly conserved regions (Figure 1D) similar to the acidic strings that are found in a subset of transcription and DNA repair factors and are known to interact with p62 PH-D of TFIIH (Figure 1E). p62 PH-D is invisible in the cryo-EM structure of apo TFIIH (19,20); however, we recently modelled the structure of human TFIIH including p62 PH-D, and those of TFIIH bound to the transcription factors TFIIEα, p53 and DP1, and the NER factors XPC and UVSSA (Figure 1F) (30).

**Figure 2.**
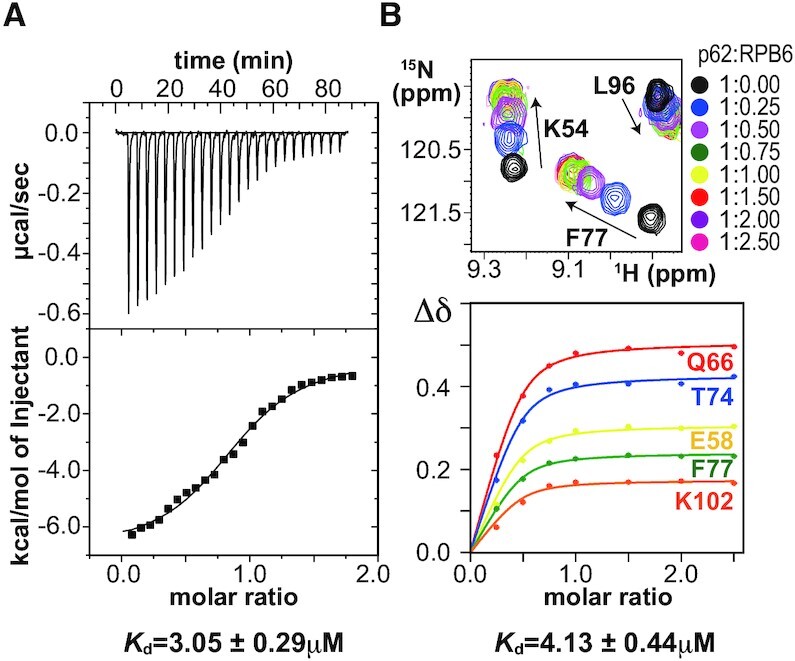
The short tail common to RNAPs has binding affinity for TFIIH p62 PH-D. (**A**) ITC analysis of p62 PH-D binding by RPB6 (residues 1–24). (**B**) NMR analysis of p62 PH-D binding by full-length RPB6. Overlay of ^1^H–^15^N HSQC spectra of p62 titrated with RPB6 and titration curves.

Thus, we examined whether vertebrate RPB6 NTT indeed binds to p62 PH-D. ITC experiments indicated that human p62 PH-D binds to the NTT peptide derived from human RPB6, but not from yeast Rpb6 (Figure 2A and Supplementary Figure S2). Concordantly, an NMR titration experiment revealed that human p62 PH-D binds specifically to human full-length RPB6 in a 1:1 stoichiometric ratio (Figure 2B and Supplementary Figure S3).

### RPB6 catches the PH-D of TFIIH p62 by its flexible NTT

**Figure 3.**
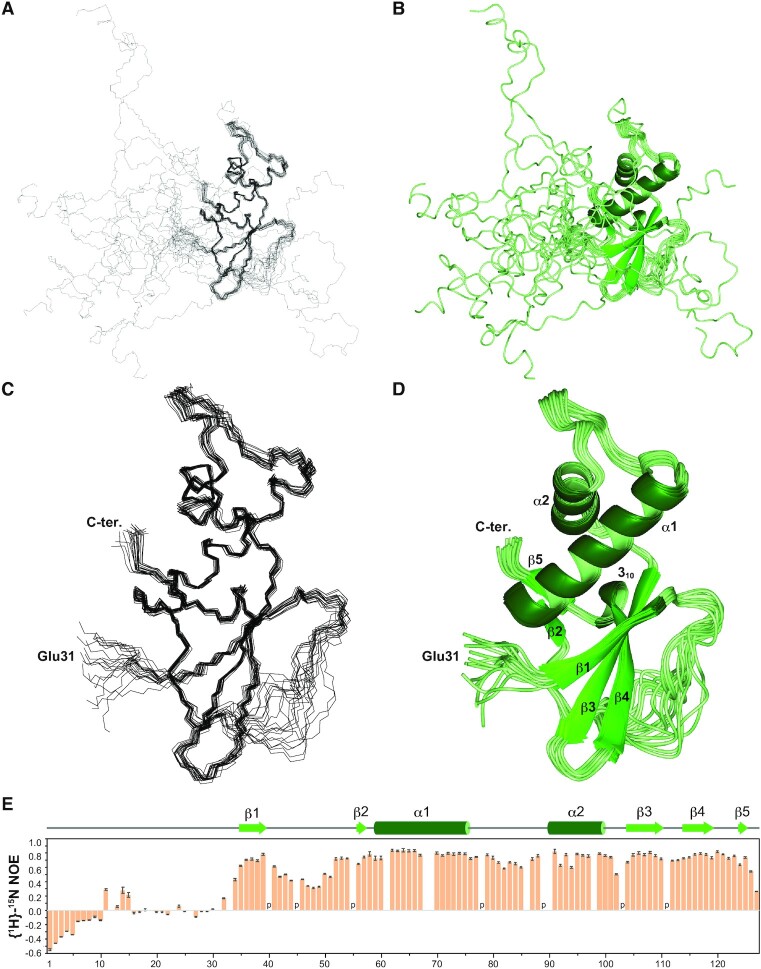
Structure and dynamics of human RPB6. (**A**, **C**) The 20 best solution structures of RPB6 shown as a line diagram. (**B**, **D**) The 20 best solution structures shown as a ribbon diagram. In (C) and **(**D), the N-terminal 30 residues are omitted for clarity. (**E**) NMR relaxation analysis. Heteronuclear {^1^H}–^15^N NOE values are plotted for each residue. Proline residues are indicated by ‘p’. For some residues, no NOE value was determined due to the overlapping of amide peaks.

Next, we determined the structure of RPB6, both unbound and bound to p62 PH-D, by NMR (Table 1). Unbound and bound RPB6 were similar to each other and to a previously reported structure (34) (Supplementary Figure S4): NTT (residues 1–32) adopts random conformations (Figure 3A–D), as confirmed by ^15^N relaxation analysis (Figure 3E); while residues 34–39, comprising the strand β1, form a β-sheet with β3 and β4.

**Figure 4.**
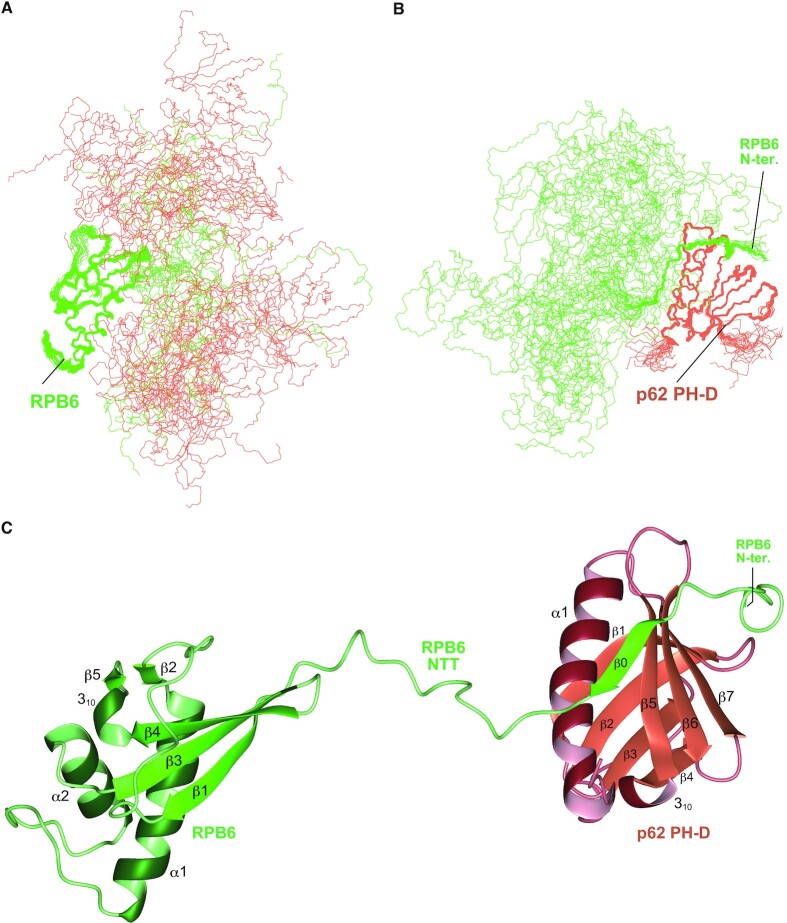
Structure of the RPB6–TFIIH p62 PH-D complex. (**A**) The 20 best solution structures of the RPB6–TFIIH p62 PH-D complex superimposed over residues 36–39 and 53–125 of RPB6. (**B**) The 20 best solution structures of the RPB6–TFIIH p62 PH-D complex superimposed over residues 3–19 of RPB6 and residues 7–104 of TFIIH p62 PH-D. (**C**) Ribbon diagram of one model of the structural ensembles. RPB6 is colored green; p62 PH-D is colored coral.

**Figure 5.**
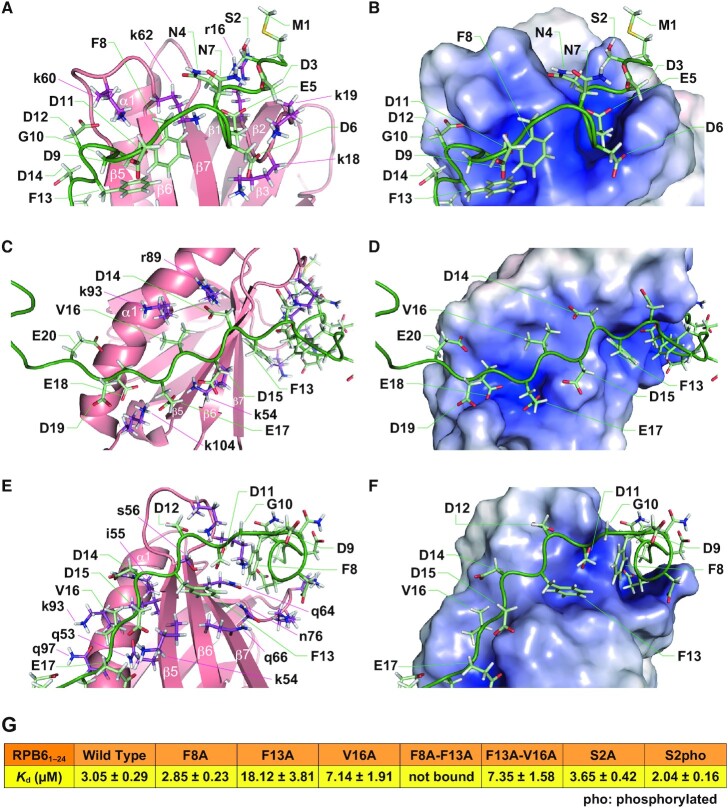
Intermolecular interactions. (A–D) Electrostatic interactions between acidic residues of RPB6 NTT and basic residues of TFIIH p62 PH-D. (**E**, **F**) Interactions between Phe8, Phe13, and Val16 of RPB6 NTT and TFIIH p62 PH-D. In (**A**, **C**, **E**), RPB6 is shown in stick representation (green/pale green) and p62 is shown in ribbon representation (coral/magenta). In (**B**, **D**, **F**), the electrostatic potential surface of p62 is shown. Positive potential is shown in blue, and negative potential in red. RPB6 is shown in stick representation (green/pale green). To discriminate p62 from RPB6, residues of p62 are labeled in lowercase and residues of RPB6 are labeled in uppercase. (**G**) *K*_d_ estimated from ITC analysis of p62 PH-D binding by mutant RPB6 (residues 1–24).

In the bound structure (Figures 4 and 5), NTT β0 (residues 14–17) interacts with PH-D β5 (Figure 4C), while Phe13 fits into a hydrophobic pocket in PH-D (Figure 5E,F) and Val16 is also buried in the PH-D groove (Figure 5C, D). In alanine substitution experiments, PH-D binding was diminished by F13A and V16A, but not by F8A (Figure 5G). While no further reduction was caused by F13A-V16A double mutation, F8A-F13A double mutation entirely abolished PH-D binding, suggesting that, in the absence of Phe13, Phe8 can fit into the pocket with reduced binding activity (Figure 5G).

RPB6 is reportedly phosphorylated at Ser2 *in vivo* (35,36). We therefore examined the effect of its phosphorylation or substitution to alanine on the interaction with p62 PH-D. ITC experiments showed that Ser2 phosphorylation or its alanine substitution had only a modest effect on PH-D binding *in vitro* (Figure 5G), concordant with the finding that RPB6 Ser2 is located outside the PH-D groove in the bound state (Figures 4 and 5).

### RPB6 NTT of RNAPII is a binding platform for holo-TFIIH

**Figure 6.**
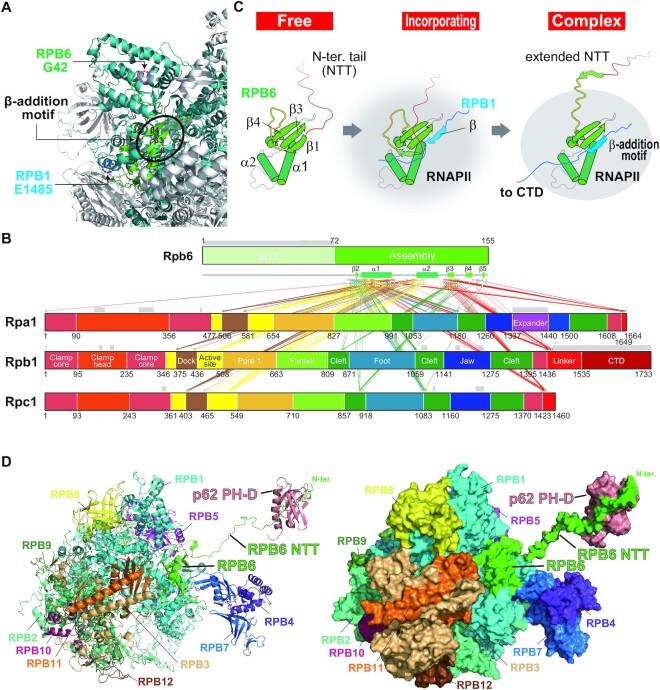
Structural model of the complex of human RNAPII and TFIIH p62 PH-D based on the extension model of RPB6 NTT. (**A**) Formation of a β-addition motif between RPB6 and RPB1 in RNAPII. Human RNAPII (PDB ID: 5IYB) is shown with colored subunits: RPB6 is shown in green, RPB1 in cyan, and the other subunits in white. In β-addition motifs, a β-strand from one protein is added to a β-sheet of another protein; in RNAPII, the last β-strand from the largest subunit RPB1 is incorporated in the β-sheet comprising strands β3 and β4 of RPB6. The most N-terminal residue of RPB6 and the most C-terminal residue of RPB1 that are visible in the structure are indicated by magenta and blue dotted circles, respectively. (**B**) Interactions between Rpb6 and the largest subunit of each RNAP from yeast. PDB ID: 5W65, RNAPI; 5FYW, RNAPII; 6EU0, RNAPIII. Residues within 5 Å are linked with lines that are color-coded according to domain structure (57–59). Invisible regions in the structure are indicated with grey horizontal bars. (**C**) Extension model of RPB6 NTT. Red indicates free RPB6 NTT; cyan indicates RPB1 β-strand. (**D**) A structural model of the human RNAPII–p62 PH-D interaction. Left, shown in ribbon representation; right, in molecular surface representation. RPB6 is colored green; p62 PH-D is colored coral.

Although β1 of free RPB6 interacts with β3 and β4 (Figure 4C), it is unfolded in the RNAPII complex (part of the invisible 41 residues; Figure 6A), in which β3 and β4 form an alternative β-sheet, called a β-addition motif, together with the β-strand in the largest subunit RPB1 that precedes the invisible CTD. This β-addition motif is also formed between RPB6 and RPA1, the largest subunit of RNAPI, and between RPB6 and RPC1, the largest subunit of RNAPIII (Figure 6B and Supplementary Figures S5 and S6). From these observations, we propose an extension model of RPB6 NTT (Figure 6C); when RPB6 is incorporated into RNAP, the N-terminal 10 amino acid residues of β1 is unfolded to form the extended NTT together with the 32 amino acid NTT of free RPB6. Using such an unfolded RPB6 NTT, we built a structural model of the complex of RNAPII and p62 PH-D (Figure 6D and Supplementary Figure S7). This model demonstrated that the RPB6 NTT in RNAPII is long enough for p62 PH-D to bind. In all three RNAPs, RPB6 NTT is likely to access PH-D in a similar way.

**Figure 7.**
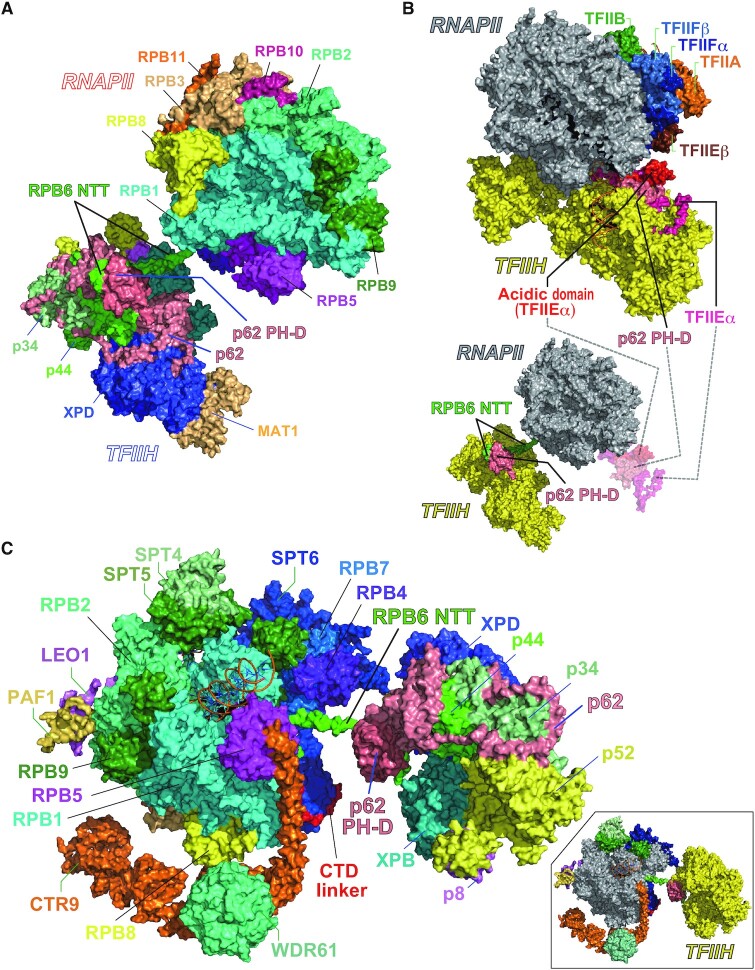
Steric hindrance may prevent the human RNAPII–TFIIH interaction during transcription initiation, but not during transcription elongation. (**A**) Structural model of the complex of human RNAPII with TFIIH. (**B**) Structure comparison. Top, the human PIC (PDB ID 6O9L); bottom, our model of the RNAPII–TFIIH complex. As a reference, TFIIEα and p62 PH-D in PIC are indicated translucently. (**C**) Structural model of the complex of RNAPII, elongation factors, and TFIIH. In molecular surface representation, RPB6 is colored green; p62 is colored coral. For clarity, the model in which the subunits of RNAPII except for RPB6 are colored gray; the subunits of TFIIH except for p62 PH-D are colored yellow is shown in (B) and the inset in (C).

As mentioned above, we recently established a structural model of human TFIIH including p62 PH-D (30). On the basis of this model and the aforementioned model, we built a docking model of RNAPII bound to TFIIH and confirmed that RPB6 NTT is accessible to p62 PH-D in the context of TFIIH (Figure 7A and Supplementary Figure S8A). During transcription initiation, the interaction between RNAPII and TFIIH modelled here (Figure 7A and Figure 7B, bottom) is unlikely to occur because TFIIEα closely interacts with p62 PH-D at this point (37) (Figure 7B, top and Supplementary Figure S8B). However, elongating RNAPII is likely to interact with TFIIH without steric hindrance, as modelled from the cryo-EM structure of RNAPII surrounded by the elongation factors DSIF (SPT4 and SPT5), PAF (PAF1, LEO1, CTR9, CDC73 and WDR61) and SPT6 (38) (Figure 7C and Supplementary Figure S9).

### RPB6 binding to p62 through the NTT is critical for cell growth and transcription by the three RNAPs

**Figure 8.**
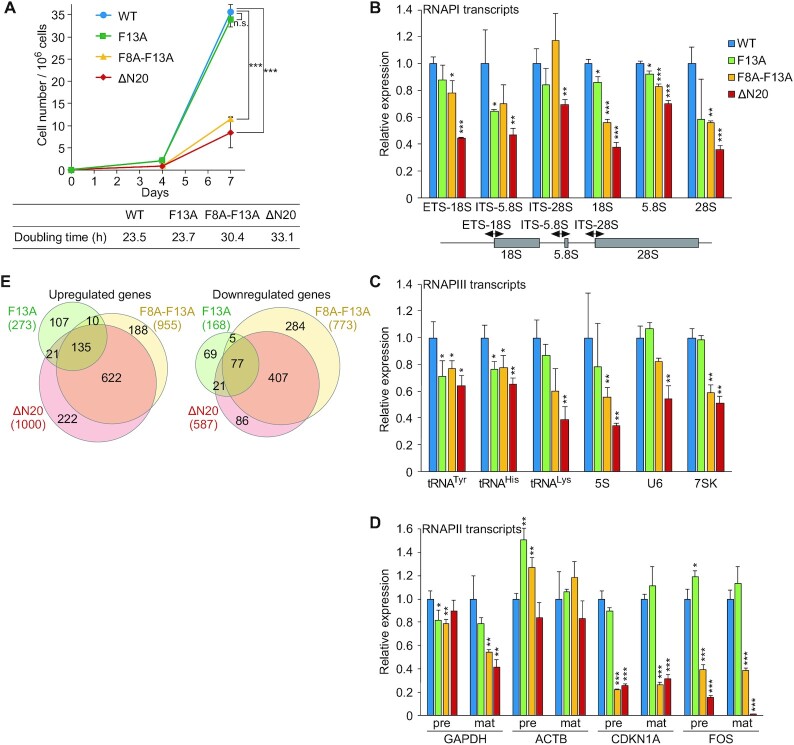
Role of the interaction between TFIIH-p62 and RPB6 in cell growth and transcription. (**A**) Growth curves of HeLa cells expressing WT or mutant RPB6. Cell numbers were counted at days 0, 4 and 7. Doubling time is indicated in the table below. Data represent mean ± S.D. (*n* = 3). n.s., not significant; ***P < 0.001 (two-tailed Student's *t*-test). (B–D) qRT-PCR analysis of RNAPI (**B**) RNAPIII (**C**) and RNAPII (**D**) transcripts. Pre and mat indicate precursor and mature transcripts, respectively. Data represent mean ± S.D. (*n* = 3). n.s., not significant; *P < 0.05; **P < 0.01; ***P < 0.001 (two-tailed Student's *t*-test). (**E**) Venn diagram showing upregulated (left) and downregulated (right) genes relative to WT, determined by RNA-seq analysis (FC > 2; FDR *q*-value < 0.05).

To elucidate the biological significance of this interaction, we knocked down endogenous RPB6 expression and re-expressed wild-type (WT) or mutant RPB6 in HeLa cells using lentivirus vectors (Figure 8A and Supplementary Figure S10A–D). Knockdown of RPB6 resulted in cell death, but simultaneous expression of RNAi-resistant RPB6 WT restored cell viability. Simultaneous expression of F8A-F13A or ΔN20 RPB6 did not fully restore cell growth, suggesting that the p62–RPB6 interaction is critical for cell proliferation.

To investigate the underlying cause of growth defects in F8A-F13A and ΔN20 cells, transcript levels were compared among RNAPI-, RNAPII-, and RNAPIII-transcribed genes. We found that both precursor and mature rRNAs were modestly downregulated (Figure 8B), and some RNAPIII-transcribed genes also showed reduced expression in F8A-F13A and ΔN20 cells (Figure 8C), indicating that the p62–RPB6 interaction is involved in both RNAPI and RNAPIII transcription. Regarding RNAPII-transcribed genes, although mutations in RPB6 NTT had little effect on *ACTB* expression, *CDKN1A* and *FOS* expression was severely attenuated in F8A-F13A and ΔN20 cells (Figure 8D), suggesting that the p62–RPB6 interaction is important for RNAPII transcription in a gene-specific manner. Given these findings, we performed RNA-seq analysis (Figure 8E). There was significant overlap among the genes that were up- or downregulated in F13A, F8A-F13A and ΔN20 cells; in particular, genes associated with cell adhesion were enriched in the commonly upregulated genes (Supplementary Figure S10E). However, protein-coding genes related to NER were not substantially affected in F8A-F13A or ΔN20 cells (Supplementary Figure S10F).

### RPB6 binding to p62 through the NTT is critical for TC-NER of RNAPI- and RNAPII-transcribed genes

**Figure 9.**
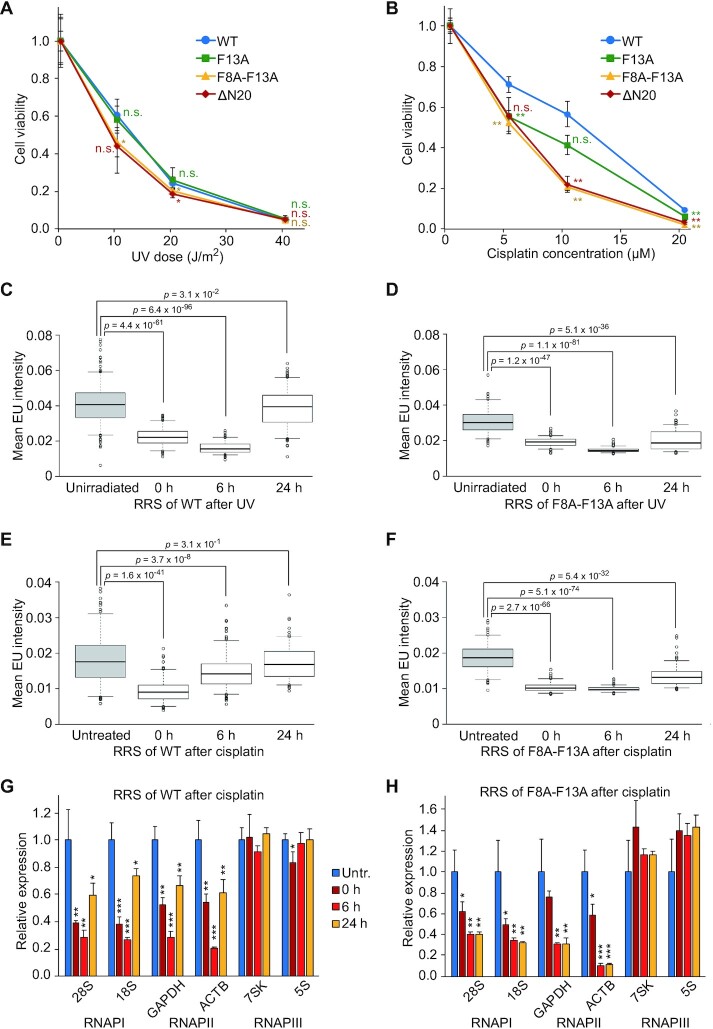
Role of the interaction between TFIIH-p62 and RPB6 in TC-NER. (**A**) Survival of HeLa cells after UV-C irradiation. Cell numbers were counted 72 h after UV-C irradiation. Data represent mean ± S.D. (*n* = 3). n.s., not significant; *P < 0.05 (two-tailed Student's *t*-test). (**B**) Survival of HeLa cells after cisplatin treatment. Cell numbers were counted after 72 h of incubation with cisplatin. Data represent mean ± S.D. (*n* = 3). n.s., not significant; **P < 0.01 (two-tailed Student's *t*-test). (C–F) RRS in HeLa cells after UV-C irradiation at 40 J/m^2^ (**C** and **D**) or treatment with 100 μM cisplatin for 2 h (**E** and **F**). Nascent RNA was labeled with EU and visualized with Alexa Fluor 488-azide, and mean fluorescence intensity was determined for at least 125 cells per condition. *P*-values were calculated by two-tailed Student's *t*-test. Whiskers indicate the 5th and 95th percentiles, and dots represent outliers. (**G** and **H**) RRS of individual genes after cisplatin treatment. Data represent mean ± S.D. (*n* = 3). n.s., not significant; *P < 0.05; **P < 0.01; ***P < 0.001 (two-tailed Student's *t*-test).

Because TFIIH plays an essential role in the DNA opening step of NER (39,40), we investigated DNA repair proficiency by measuring cell survival after UV irradiation or cisplatin treatment. These types of DNA damage are primarily repaired through the NER pathway (41). As compared with WT cells, F8A-F13A and ΔN20 cells were more sensitive to UV-C and cisplatin exposure (Figure 9A, B). To differentiate between the TC and global genome (GG) NER pathways, which differ in initial damage recognition, we used fluorescence-based assays to measure recovery of RNA synthesis (RRS) and unscheduled DNA synthesis (UDS), which respectively reflect TC-NER and GG-NER (31). In WT cells, RNA synthesis decreased immediately after UV-C irradiation or cisplatin treatment, but recovered almost completely within 24 h (Figure 9C, E and Supplementary Figure S11). In F8A–F13A cells, however, inhibition of RNA synthesis persisted (Figure 9D, F), suggesting that TC-NER is defective in F8A–F13A cells. By contrast, WT and F8A–F13A cells showed similar UDS after UV-C irradiation or cisplatin treatment (Supplementary Figure S12), suggesting that the p62–RPB6 interaction is selectively involved in TC-NER. Further analysis (Supplementary Figures S13 and S14) indicated that the p62–RPB6 interaction plays independent roles in transcription and NER, and that both contribute to cell growth (Supplementary Text).

In the microscopy-based RRS assay, rRNA synthesis was the main source of the signal (Figure 9C–F and Supplementary Figures S11). Given that RPB6 is common to all three RNAPs, we investigated RRS of genes transcribed specifically by each RNAP by qRT-PCR using EU-labeled nascent RNA purified from streptavidin beads. Under the conditions employed, cisplatin treatment significantly affected RNAPI and RNAPII transcription, but not RNAPIII transcription (Figure 9G, H), probably due to the short lengths of the RNAPIII-transcribed genes. The transcriptional defects observed for RNAPI and RNAPII genes were partially recovered within 24 h of cisplatin treatment in WT, but not in F8A-F13A cells (Figure 9G, H), suggesting that the p62–RPB6 interaction is critical for TC-NER of RNAPI- and RNAPII-transcribed genes.

### RPB6 NTT in yeasts

So far, we have shown that human RPB6 NTT interacts with PH-D of the p62 subunit of human TFIIH. Like the human counterpart, yeast Rpb6 core forms the base of the clamp near the active site whereas its NTT (the N-terminal 71 residues in budding yeast, 59 residues in fission yeast) is invisible in the crystal structure of RNAPII (33,42). Apparently, most bacterial ω and archaeal RpoK, which are homologous to eukaryotic RPB6, lack NTT, whereas in yeasts the NTT has only limited sequence similarity to the vertebrate counterparts (Figure 1D) (43). Thus, whether yeast Rpb6 NTT binds to PH-D of Tfb1, the yeast counterpart of human p62, and plays a role in transcription or DNA repair is an interesting question that needs further study. Despite low sequence similarity, acidic amino acids are abundant with a phenylalanine sandwiched between them in the first half of NTT in yeasts. Meanwhile, the binding surface of p62 PH-D is not highly conserved between humans and yeasts (7,14), leading us to speculate a different binding mode between yeast Rpb6 NTT and Tfb1 PH-D. In yeasts, Rpb6 is essential for survival and genetically interacts with other subunits of RNAPs and various factors such as TFIIS, a proteosome subunit, 5'-3' exoribonuclease, and trimethylguanosine synthase (44–51). Notably, the N-terminal 42 amino acids of budding yeast Rpb6 and the N-terminal 60 amino acids of fission yeast Rpb6 are dispensable for survival (44,46). In one study, however, out of 14 Rpb6 temperature-sensitive mutants isolated in fission yeast, seven carried mutations in NTT (46), suggesting that NTT plays an important role at least at an elevated temperature in yeasts.

## CONCLUSION

It remains under debate whether TC-NER occurs during RNAPI transcription (52,53). Our finding supports its existence and suggests that TFIIH recruitment to transcription sites through the p62–RPB6 interaction is a common mechanism for TC-NER of RNAPI- and RNAPII-transcribed genes.

In a previous study, we showed that UVSSA, a key TC-NER factor involved in the recruitment of TFIIH (54,55), also interacts with p62’s PH-D (15). This interaction is mediated by UVSSA’s intrinsically disordered region spanning amino acids 400–418 and the p62’s PH-D (15). Since p62’s PH-D should not be able to bind to UVSSA and RPB6 simultaneously, how each of these interactions contributes to TC-NER is an interesting question that needs to be addressed in the future. One hypothesis is that the two interactions contribute to different steps in TC-NER. More specifically, the interaction with UVSSA may facilitate the recruitment of TFIIH to damaged sites, whereas the interaction with RPB6 may facilitate a subsequent step by tethering TFIIH to damaged sites. In support, Nakazawa *et al.* have shown that UVSSA is monoubiquitinated at K414 during DNA repair and that this monoubiquitination is critical for DNA damage-induced interaction between TFIIH and elongating RNAPII and for TC-NER (55). Thus, monoubiquitination of UVSSA K414 may trigger dissociation of p62 and its transfer to RPB6. Moreover, while *Drosophila* lacks CSA, CSB, and UVSSA homologs, it performs TC-NER (56). Given the evolutionary conservation of RPB6’s NTT among vertebrates and *Drosophila* (Figure 1D), it may be that in *Drosophila*, RPB6 interacts with p62 and plays a more important role in TC-NER as a surrogate for UVSSA.

Overall, we have shown that the common RPB6 tail in all three RNAPs (RNAPI, RNAPII and RNAPIII) plays a crucial role in recruiting TFIIH via its interaction with the PH domain of the TFIIH p62 subunit; this interaction connecting between TFIIH and all three RNAPs plays multiple roles in transcription, TC-NER, and cell proliferation. Our findings provide a plausible answer for the simple and long-standing question of why RNAPs share the RPB6 subunit with a short tail, and represent a significant conceptual advance over previous studies on transcription and TC-NER, demonstrating that TFIIH is involved extensively in multiple transcription and TC-NER systems.

## DATA AVAILABILITY

Genome Maps is an open source collaborative initiative available in the GitHub repository (https://github.com/compbio-bigdata-viz/genome-maps).

Atomic coordinates of RPB6 and RPB6–p62 PH-D complex have been deposited with the Protein Data Bank (http://www.rcsb.org) under accession number 7DTH and 7DTI, respectively, and chemical shifts and heteronuclear {^1^H}–^15^N NOE values only for RPB6 have been deposited in the BioMagResBank (BMRB) under accession number 36405 and 36406, respectively. RNA-seq data are available at Gene Expression Omnibus (GEO) (accession no. GSE166414).

## Supplementary Material

gkab612_Supplemental_File

## Notes

Present address: Hidefumi Suzuki, Graduate School of Medical Science, Yokohama City University, 3-9 Fukuura, Kanazawa-ku, Yokohama 216-0004, Japan.
