# Supplementary material for: Three human RNA polymerases interact with TFIIH via a common RPB6 subunit
Source: Nucleic Acids Res. 2021 Jul 16;50(1):1–16. doi: 10.1093/nar/gkab612 (PMC8754651; doi:10.1093/nar/gkab612)
Supplement: gkab612_Supplemental_File [file gkab612_supplemental_file.pdf]

Supplementary Text, Figures & Table

Three human RNA polymerases interact with TFIIH via a common RPB6 subunit

Masahiko Okuda[1,†], Tetsufumi Suwa[2,†], Hidefumi Suzuki[2], Yuki Yamaguchi[2], and Yoshifumi Nishimura[1,3]

[1] Graduate School of Medical Life Science, Yokohama City University, 1-7-29 Suehiro-cho, Tsurumi-ku, Yokohama 230-0045, Japan

[2] School of Life Science and Technology, Tokyo Institute of Technology, Yokohama, 226-8501, Japan

[3] Graduate School of Integrated Sciences for Life, Hiroshima University, 1-4-4 Kagamiyama, Higashi-Hiroshima 739-8258, Japan

[†] The authors wish it to be known that, in their opinion, the first two authors should be regarded as Joint First Authors.

Present Address: Hidefumi Suzuki, Graduate School of Medical Science, Yokohama City University, 3-9 Fukuura, Kanazawa-ku, Yokohama 216-0004, Japan

Supplementary Text

Causality among transcription, NER, and growth defects in F8A-F13A and ΔN20 cells

We considered whether the transcriptional defects in F8A-F13A and ΔN20 cells resulted in NER defects. Because protein-coding genes related to NER were not substantially affected in F8A-F13A or ΔN20 cells (Supplementary Figure S10F), RNAPI and RNAPIII inhibitors were used to recapitulate the transcriptional defects in these cells. Partial inhibition of RNAPI transcription with BMH21 had little effect on cell growth or cisplatin sensitivity (Supplementary Figure S13A-C). Similarly, partial inhibition of RNAPIII transcription with ML60218 only modestly inhibited cell growth and conferred cisplatin resistance, contrary to our expectation (Supplementary Figure S13D-F), suggesting that the transcriptional defects in F8A-F13A and ΔN20 cells are not the cause of NER defects.

We also explored the opposite possibility – namely, that failure to repair reactive oxygen species (ROS)-induced DNA lesions might result in growth defects and transcriptional defects. Prior treatment with N-acetyl-L-cysteine (NAC), a commonly used antioxidant, partially restored the growth defects in F8A-F13A cells, but had little effect on the growth of WT cells (Supplementary Figure S14A). By contrast, NAC had no discernible effect on RNAPI, RNAPII, or RNAPIII transcription in either WT or F8A-F13A cells, except for a few exceptions (Supplementary Figure S14B-D), suggesting that NER defects only partially account for the growth defects observed in F8A-F13A cells. It is therefore plausible that the p62–RPB6 interaction functions independently in transcription and NER, and that both functions contribute to cell growth.

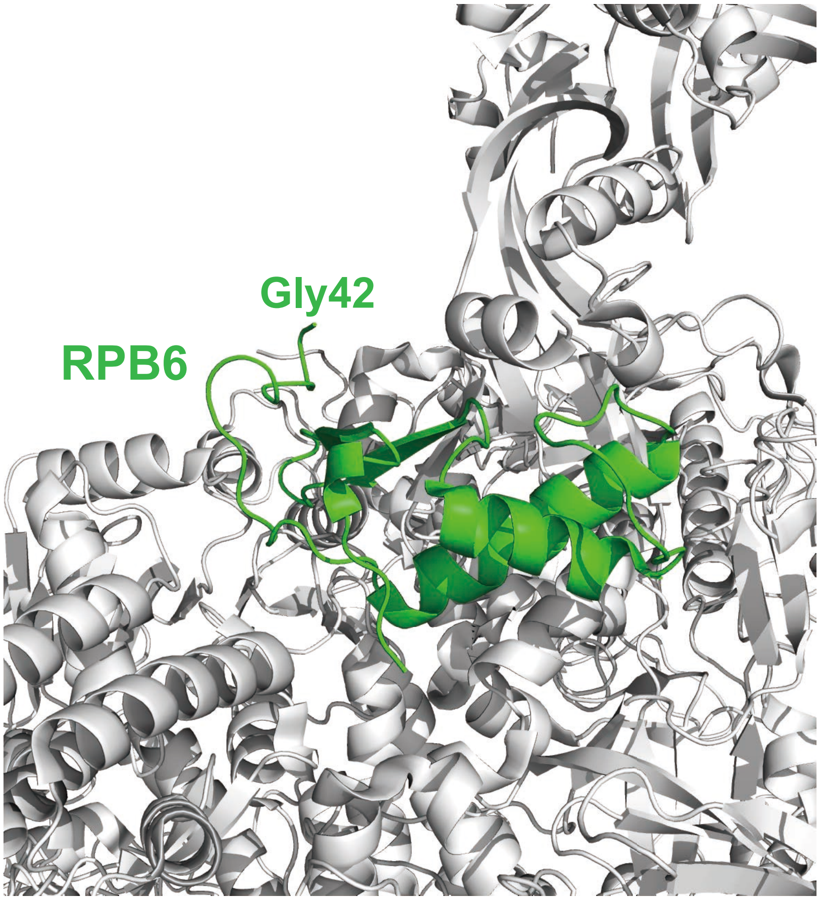

Supplementary Figure S1. RPB6 in human RNAPII (PDB ID: 5IY6).
RPB6 is shown in green and the other subunits are colored in gray. The N-terminal 41 residues of RPB6 are invisible.

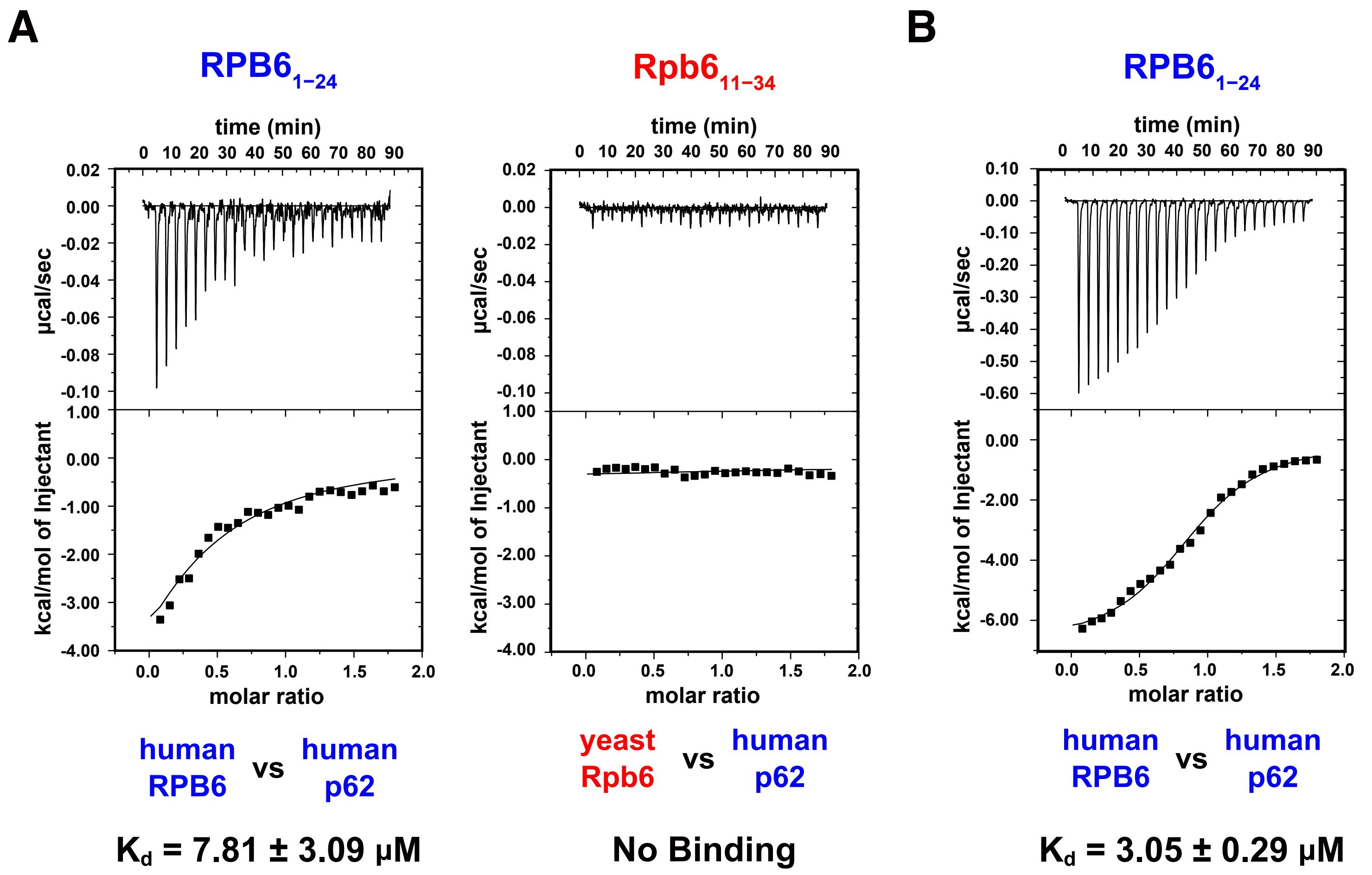

Supplementary Figure S2. ITC analysis of TFIIH p62 PH-D by RPB6.
Shown are thermograms (upper panels) and binding isotherms (lower panels) of the calorimetric titration. The K_d values of the interaction between human p62 PH-D and human RPB6 (residues 1−24+Tyr) or yeast Rpb6 (residues 11−34) were measured by ITC using a VP-ITC calorimeter (MicroCal).
(**A**) Calorimetric titration between 100 µM human RPB6 or yeast Rpb6 in the syringe (25×20 µL injections) and 2 mL of 10 µM human p62 PH-D in the cell was carried out in 20 mM potassium phosphate (pH 6.8) at 20°C. Each injection took 4 s, with a pre-injection delay of 210 s and a syringe stirring speed of 307 rpm. Data were analyzed by using the Origin software package (MicroCal).
(**B**) Calorimetric titration between 300 µM human RPB6 in the syringe (25×20 µL injections) and 2 mL of 30 µM human p62 PH-D.

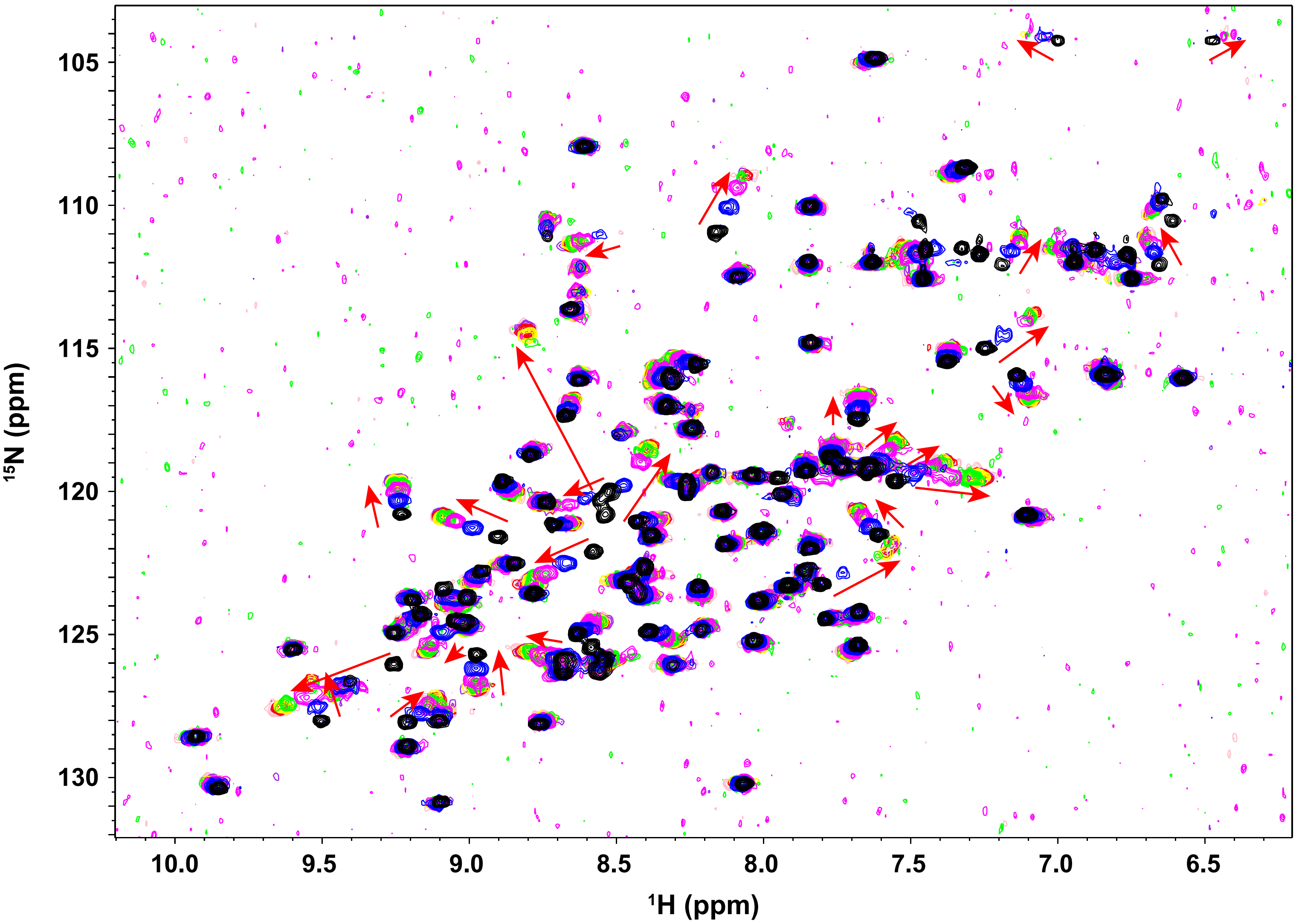

Supplementary Figure S3. NMR analysis of p62 PH-D binding by RPB6.
Shown are overlay of selected region of ^{1}H–^{15}N HSQC spectra of p62 PH-D before [black] and after the addition of full-length RPB6, residues 1–127 [blue 1: 0.25, magenta 1: 0.50, green 1: 0.75, yellow 1: 1.00, red 1: 1.50, purple 1: 2.00 and pink 1: 2.50].

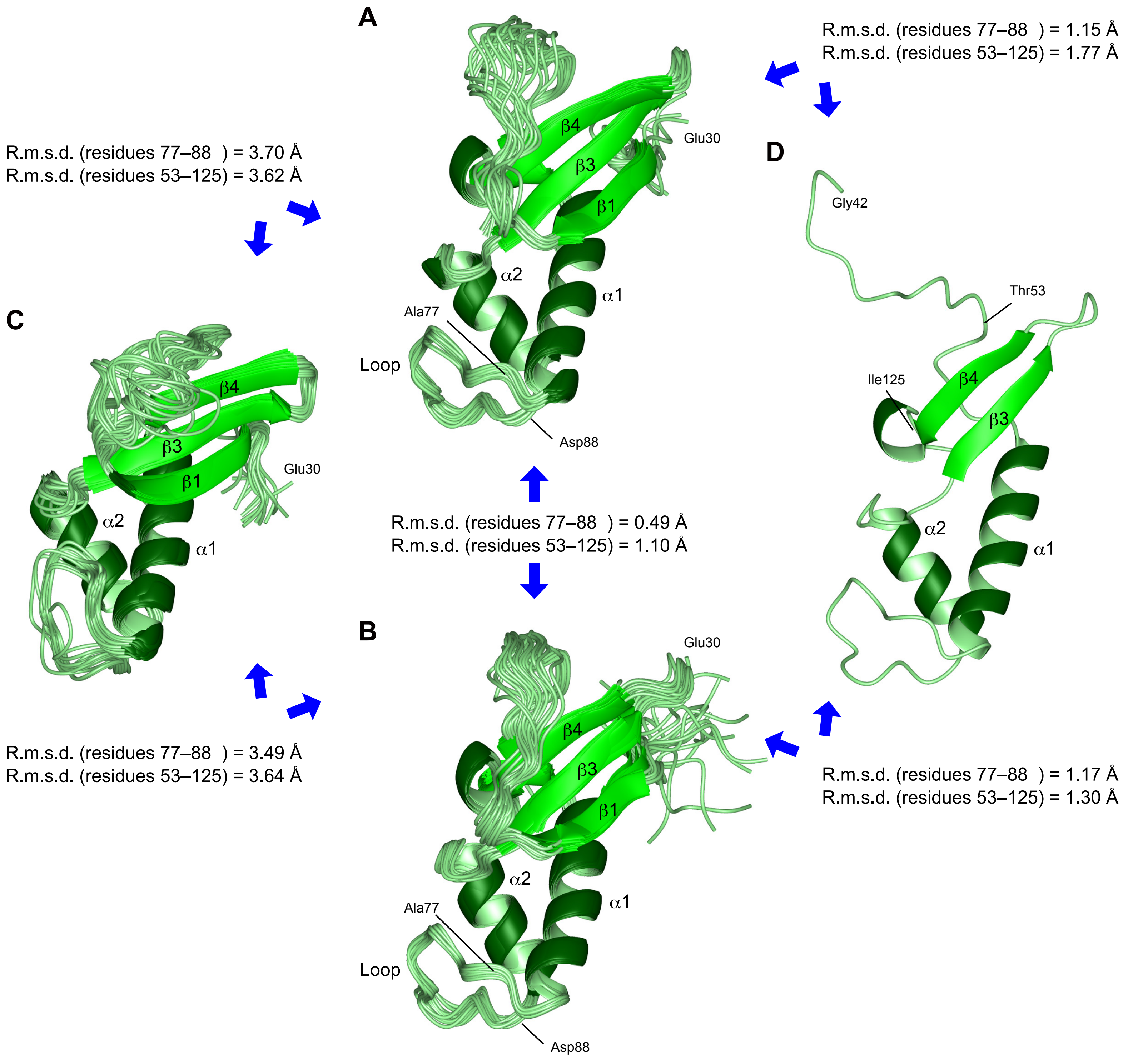

Supplementary Figure S4. Structures of RPB6 in a free form and in complex of RNAPII.
(**A**) NMR solution structures of RPB6 in a free form, determined in this study (PDB ID: 7DTH)
(**B**) NMR solution structures of RPB6 in a bound form, determined in this study (PDB ID: 7DTI).
(**C**) NMR solution structures of RPB6 in a free form (PDB ID: 1QKL).
(**D**) Cryo-EM structure of RPB6 in complex of RNAPII (PDB ID: 5IYB).
R.m.s. deviations of the backbone atoms for the loop region (residues 77–88) and the overlapped region (residues 53–125) are indicated below.

Although our structures (A,B) are similar to the one (C) determined in earlier study; the N-terminal region, residues 1–32, is totally disordered and the following β1 strand is actually formed, with respect to the region of residues 53–125 they are more similar to the crystal and cryo-EM structures of RPB6 in RNAPII (D). Particularly, conformation of a loop connecting helices α1 and α2, which shows a low degree of flexibility (Figure 3E), more closely resembles those of them. Therefore, the structure of RPB6 is essentially identical in the isolated state and bound state in RNAPII except for the N-terminal region.

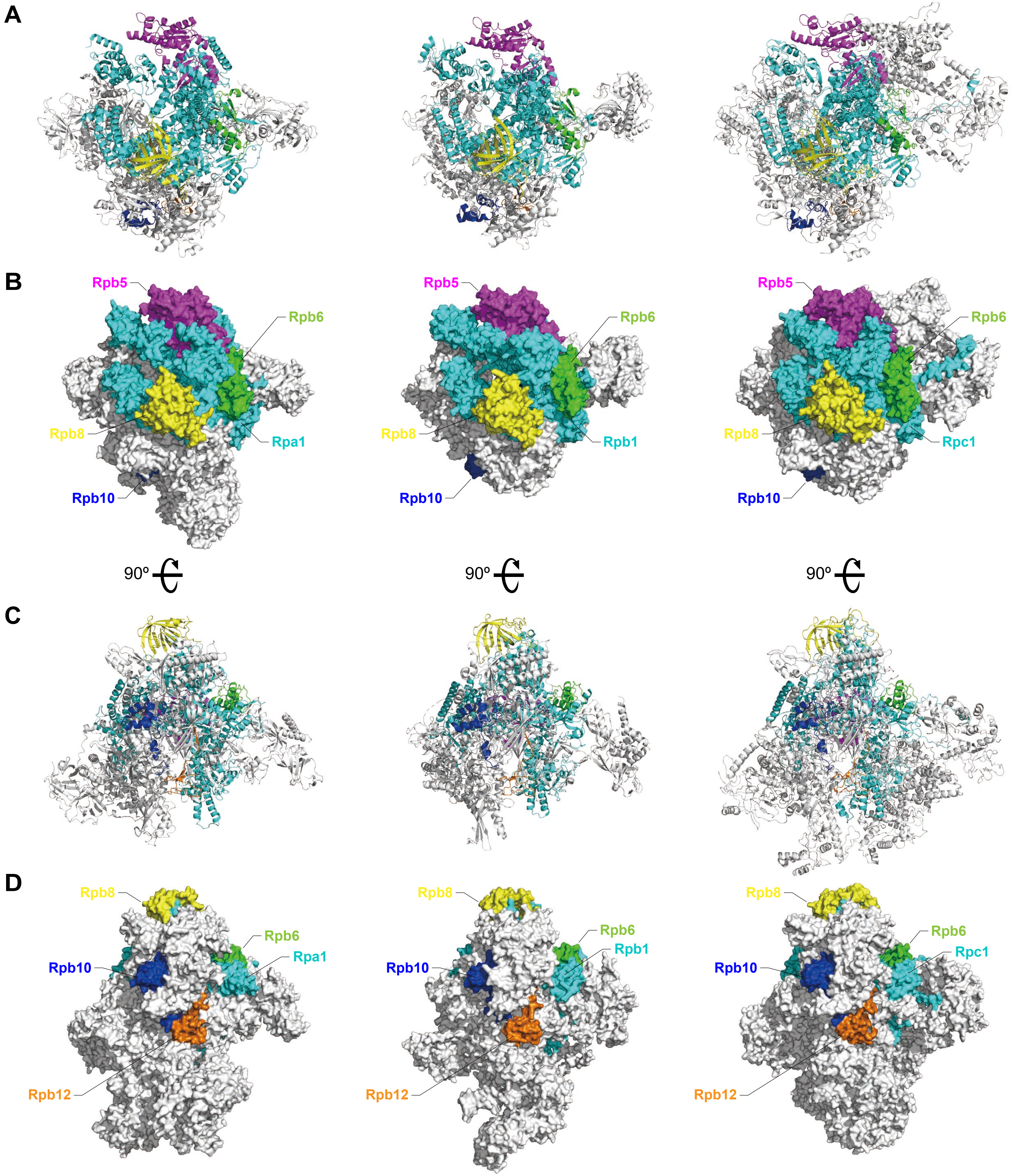

Supplementary Figure S5. The largest and the five common subunits of RNAPI, RNAPII, and RNAPIII. Ribbon (**A**,**C**) and molecular surface (**B**,**D**) representation of the structures of RNAPI (left), RNAPII (middle), and RNAPIII (right) from yeast; PDB ID: 5W65, RNAPI; 5FYW, RNAPII; 6EU0, RNAPIII. The largest subunit (Rpa1 of RNAPI; Rpb1 of RNAPII; Rpc1 of RNAPIII) is colored cyan. The five common subunits, Rpb5, Rpb6, Rpb8, Rpb10, and Rpb12 are colored magenta, green, yellow, blue, and orange, respectively. The structures in A and B are rotated by 90 degrees relative to the structures in C and D.

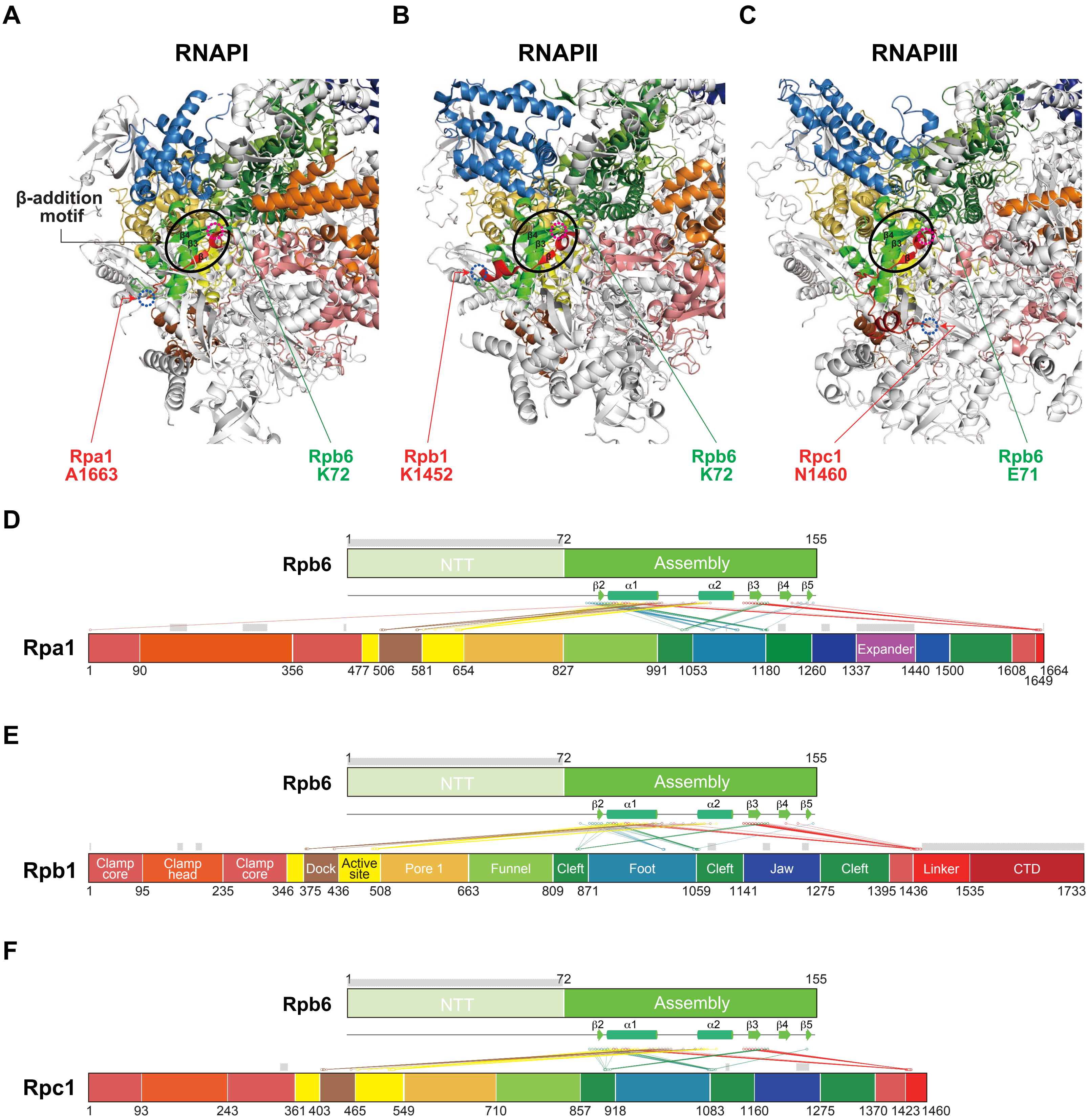

Supplementary Figure S6. Interactions between Rpb6 and the largest subunit of each RNAP from yeast. (**A**–**C**) Formation of a β-addition motif between Rpb6 and the largest subunit of each RNAP; Rpa1 of RNAPI, PDB ID: 5W65 (**A**); Rpb1 of RNAPII, PDB ID: 5FYW (**B**); Rpc1 of RNAPIII, PDB ID: 6EU0 (**C**). Rpb6 and the largest subunit of each RNAP are colored with the color code shown in (**D**–**F**). The N-terminus of Rpb6 and the C-terminus of the largest subunit of each RNAP, which are visible in the structure, are indicated by magenta and blue dotted circles, respectively.
(**D**–**F**) Interactions between Rpb6 and the largest subunit, Rpa1 of RNAPI (**D**); Rpb1 of RNAPII (**E**); Rpc1 of RNAPIII (**F**). Residues within 5 Å are linked by a line with the same color as a domain structure (57–59). Invisible regions in the structure are indicated grey dotted bars

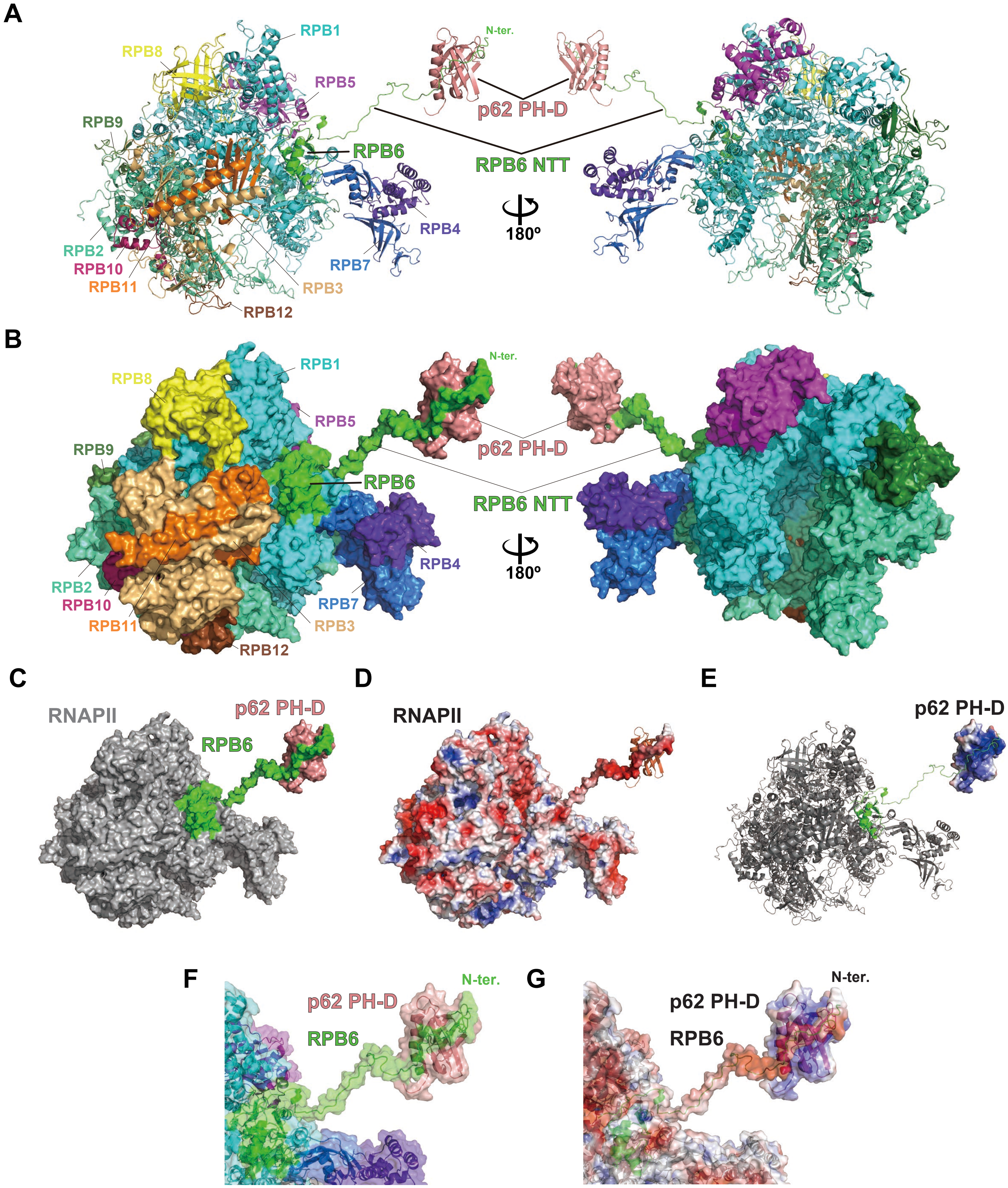

Supplementary Figure S7. Structure model of the complex of human RNAPII and TFIIH p62 PH-D.
A structural model of the human RNAPII–p62 PH-D interaction is shown in ribbon representation (**A**), molecular surface representation (**B**,**C**), electrostatic potential molecular surface (RNAPII) with ribbon (PH-D) representation (**D**), and reverse model (**E**), magnified ribbon and translucent molecular surface representation (**F**), and magnified ribbon and translucent electrostatic potential molecular surface representation (**G**). In the ribbon and molecular surface representations, RPB6 is colored green; p62 PH-D is colored coral. In the electrostatic potential molecular surface, positive and negative potentials are colored blue and red, respectively.

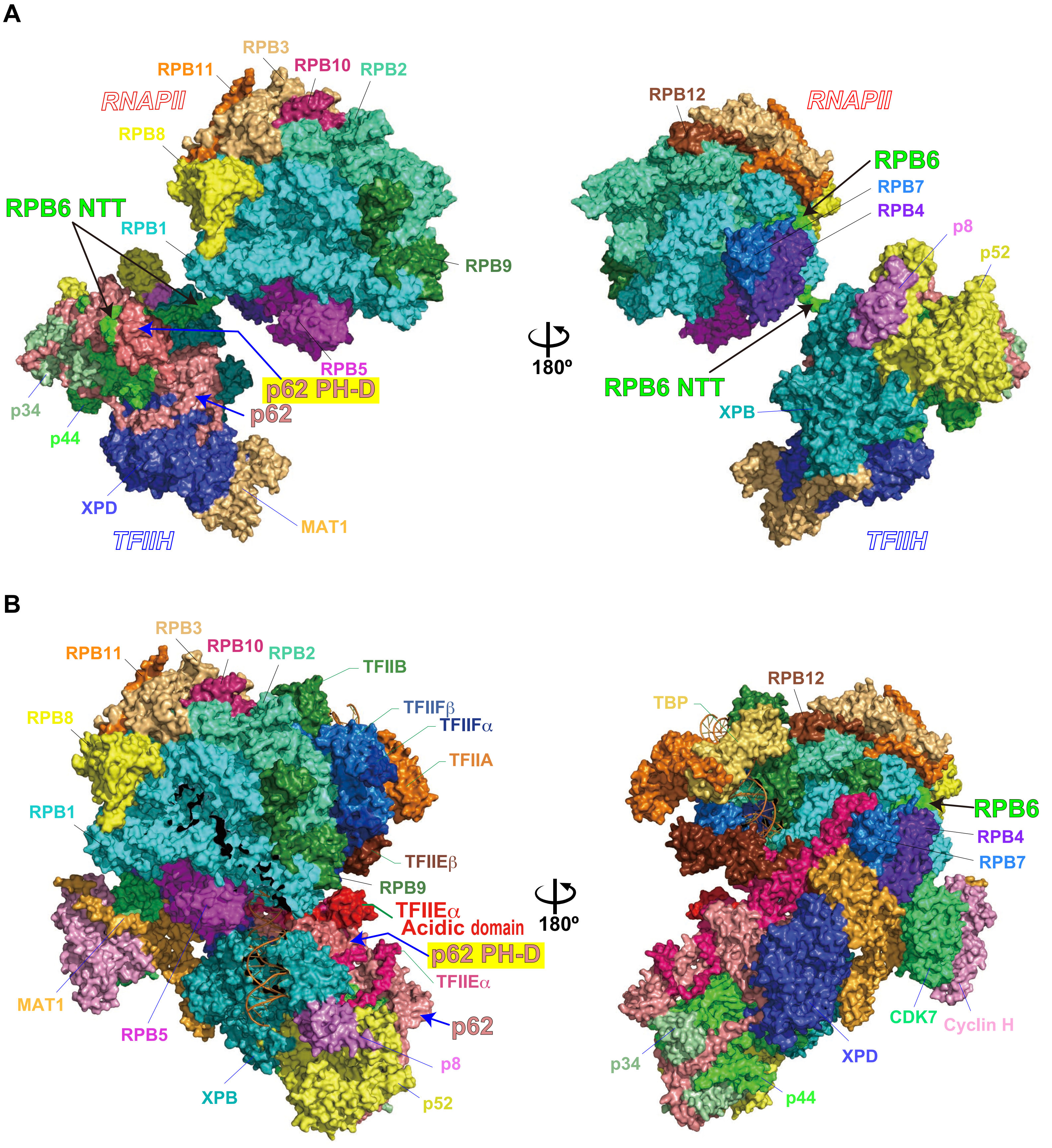

Supplementary Figure S8. Structural model of the complex of human RNAPII with TFIIH.
(**A**) Structural model of the complex of human RNAPII with TFIIH.
(**B**) Structure of the human PIC complex (PDB ID 6O9L).
In molecular surface representation, RPB6 and p62 are shown in green and coral, respectively and are indicated with arrows. The orientation of RNAPII in (A) is the same as that in (B).

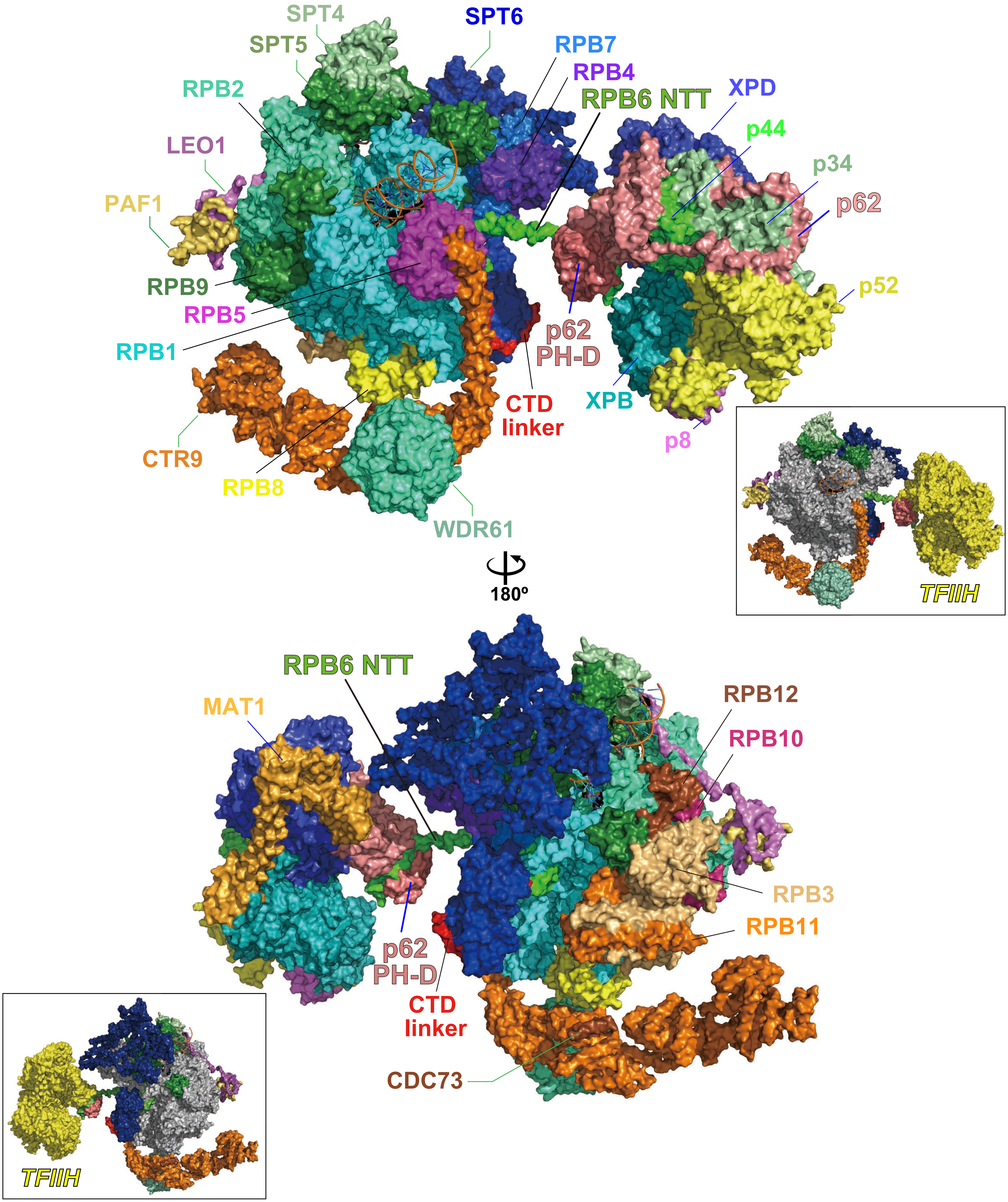

Supplementary Figure S9. Structural model of the complex of RNAPII, elongation factors and TFIIH.
In molecular surface representation, RPB6 is in green, and p62 is in coral, respectively. For clarity, the model in which the subunits of RNAPII except for RPB6 are colored gray; the subunits of TFIIH except for p62 PH-D are colored yellow is shown in the insets.

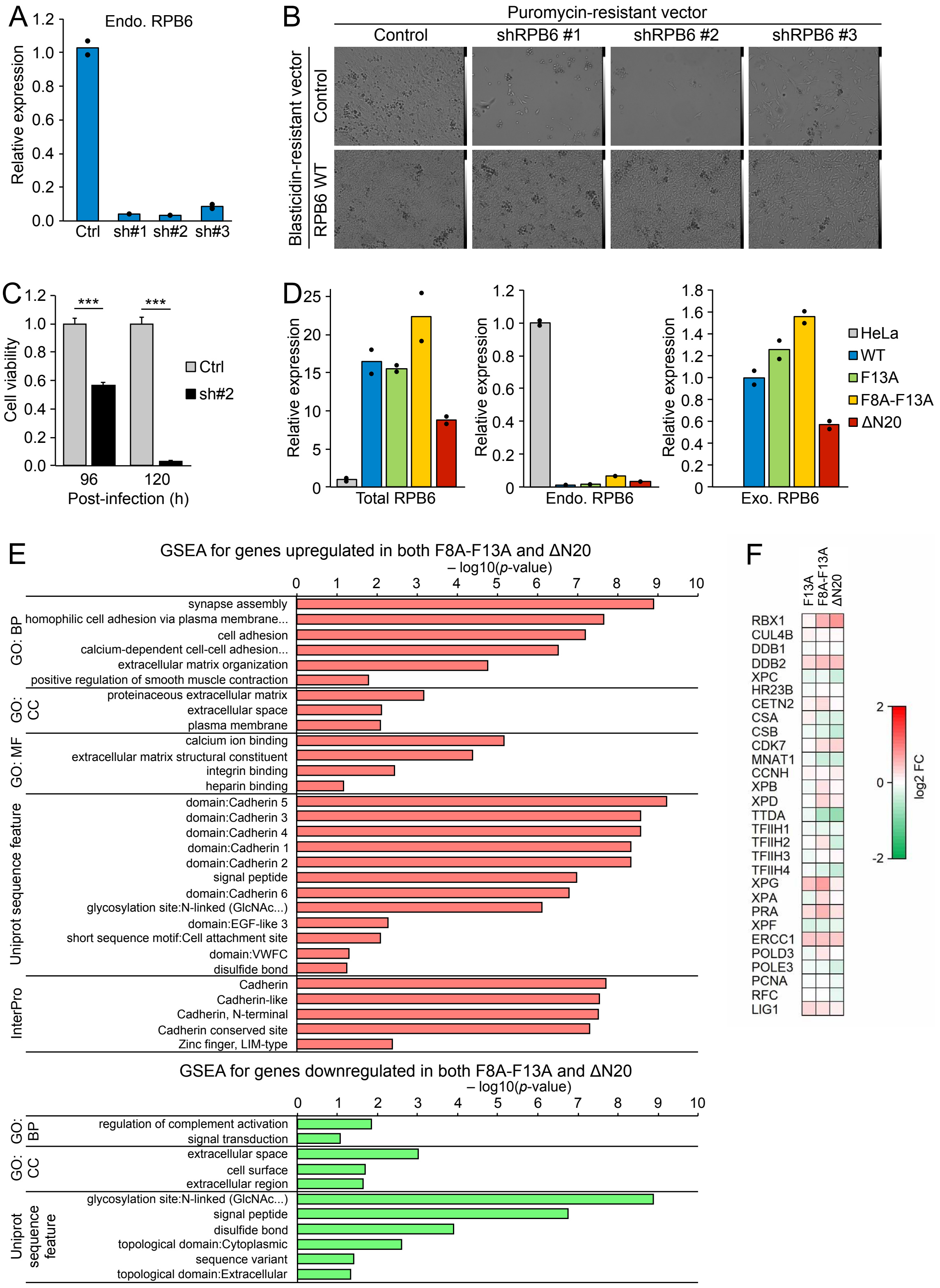

Supplementary Figure S10. Characterization of HeLa cells expressing wildtype or mutant RPB6. (**A**) qRT-PCR analysis of RPB6-knockdown HeLa cells. HeLa cells were analyzed 4 days after transduction of lentivirus vectors expressing one of shRNAs against RPB6 and puromycin selection. Data represent mean and individual data points (n = 2). (**B**) Images were taken with a phase-contrast microscope 7 days after transduction of lentivirus vectors expressing one of shRNAs against RPB6 into control or RPB6 WT-expressing cells and puromycin selection. (**C**) Cell numbers were counted 96 and 120 h after infection. Data represent mean ± S.D. (n = 3). ***, $p < 0.001$ (two-tailed Student's t-test). (**D**) qRT-PCR analysis of RPB6-knockdown HeLa cells harboring exogenous wildtype or mutant RPB6 using three primer sets that specifically detect either endogenous (Endo.) or exogenous (Exo.) RPB6 or amplify both (Total) indiscriminately. Data represent mean and individual data points (n = 2). (**E**) Gene set enrichment analysis for genes upregulated or downregulated in both F8A-F13A and ΔN20 using the Functional Annotation Tool DAVID. (**F**) Relative expression levels of genes related to NER in HeLa cells expressing RPB6 F13A, F8A-F13A, and ΔN20.

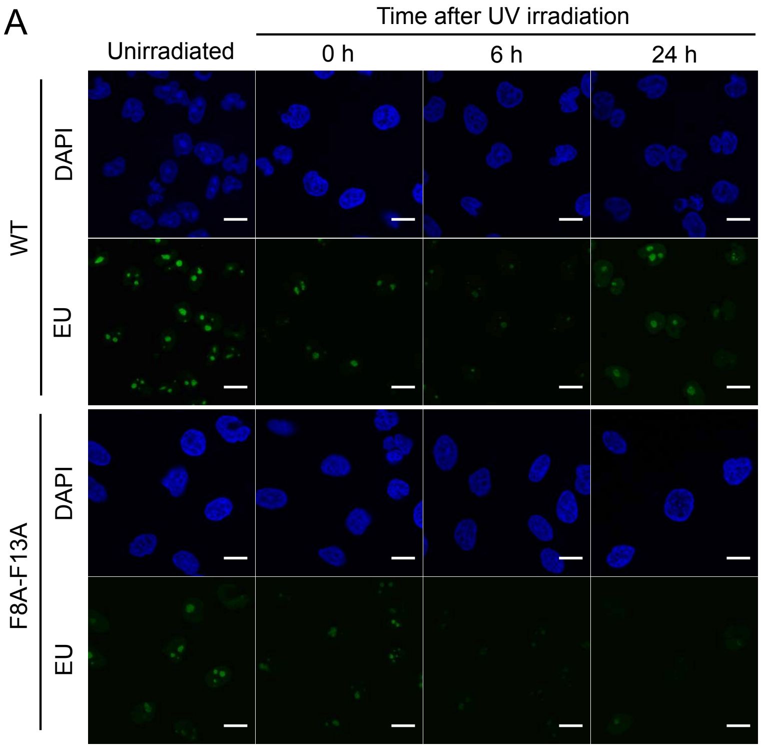

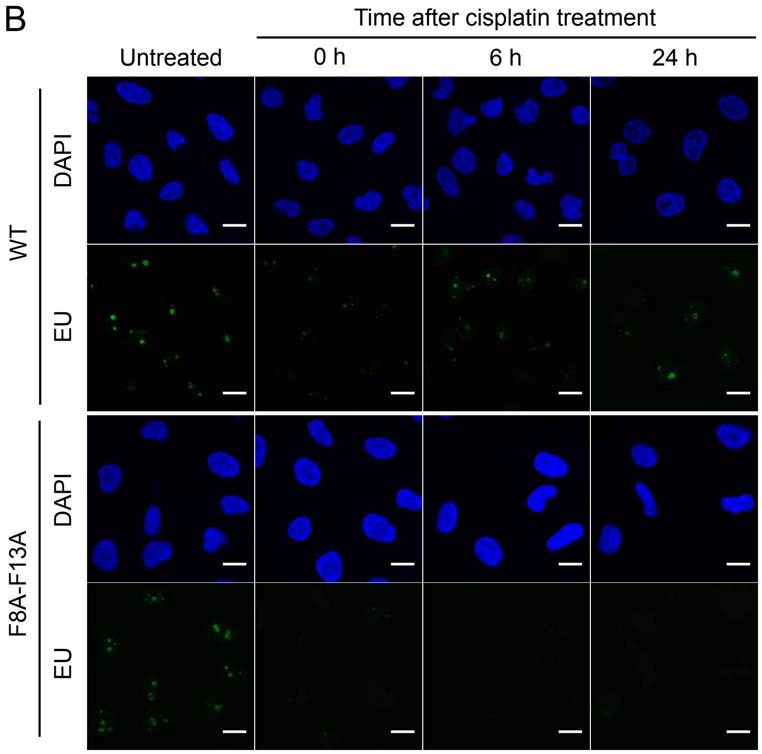

Supplementary Figure S11. Defects of TC-NER in HeLa cells expressing RPB6 F8A-F13A. (**A**) Fluorescence microscopy analysis of unirradiated cells and UV-C-irradiated cells (40 J/m^2) that were recovered for the indicated time. (**B**) Fluorescence microscopy analysis of untreated cells and cisplatin-treated cells (100 µM, 2 h) that were recovered for the indicated time. Nascent RNA was labeled with EU and visualized with Alexa Fluor 488-azide, whereas nuclei were counterstained with DAPI. Scale bar, 10 µm.

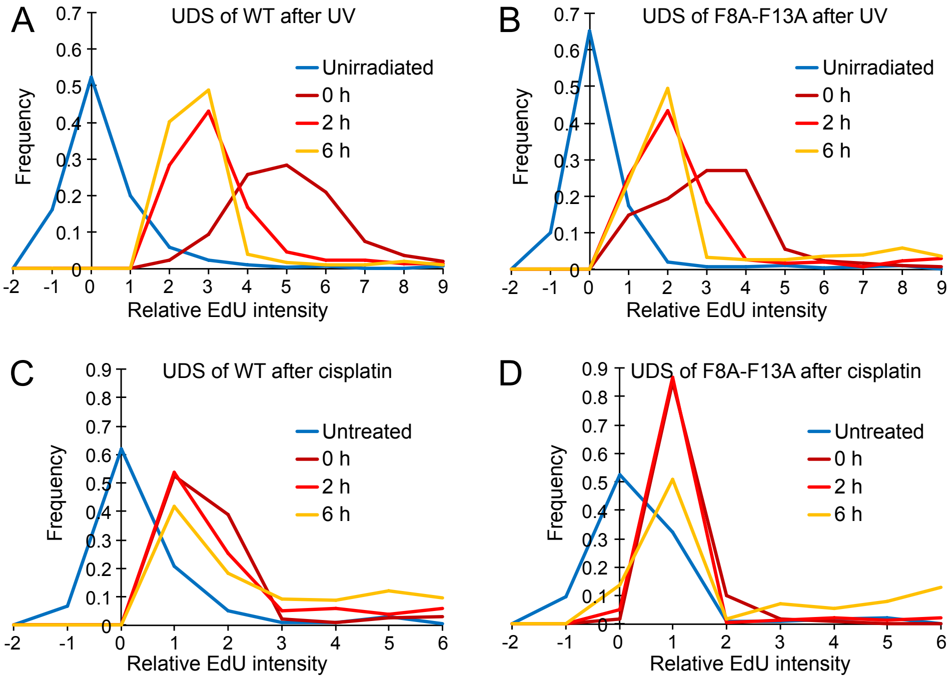

Supplementary Figure S12. UDS after UV-C irradiation or cisplatin treatment. Histograms of UDS in HeLa cells expressing RPB6 WT (**A** and **C**) or F8A-F13A (**B** and **D**) after UV-C irradiation at 40 J/m^2 (**A** and **B**) or cisplatin treatment for 2 h at 100 µM (**C** and **D**) are shown. Nascent DNA was labeled with EdU and and visualized with Alexa Fluor 488-azide. Mean fluorescence intensity of non-S-phase cells was determined for at least 250 cells per condition, log-transformed, normalized to the average intensity of control cells, and binned into a histogram.

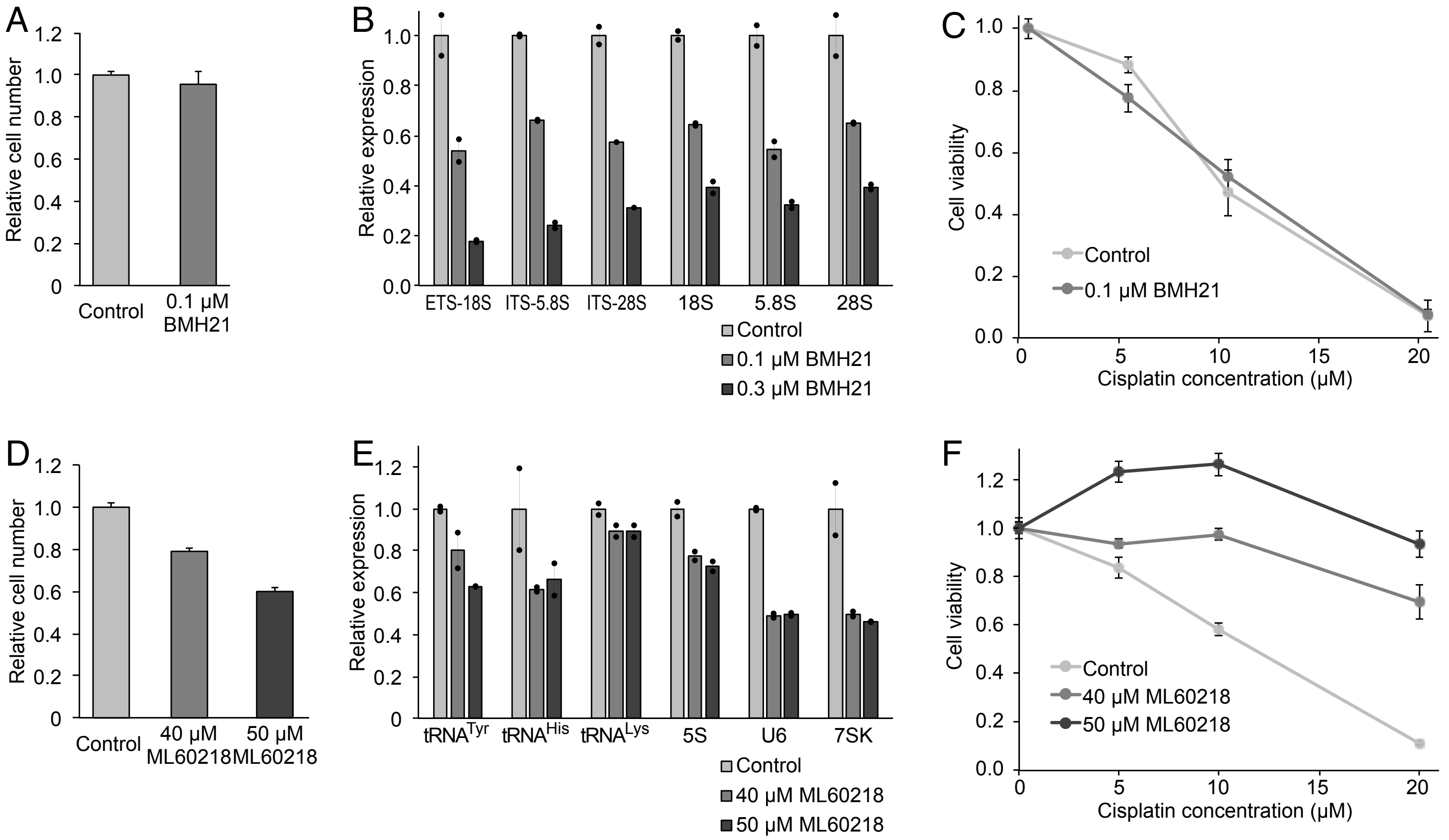

Supplementary Figure S13. Attenuation of RNAPI and RNAPIII transcription does not cause NER defects. (**A**) HeLa cells expressing RPB6 WT were incubated in the presence of the RNAPI inhibitor BMH21 or in its absence for 96 h, and then cell numbers were counted. (**B**) qRT-PCR analysis of RNAPI transcripts in WT cells incubated in the presence of BMH21 or in its absence for several days. Transcript levels were normalized to the levels in the absence of BMH21. Data represent mean and individual data points (n = 2). (**C**) Survival of WT cells after cisplatin treatment. Cell numbers were counted after 72 h of incubation with the indicated concentration of cisplatin and normalized to the numbers of untreated cells. Data represent mean ± S.D. (n = 3). (**D**) WT cells were incubated in the presence of ML60218 or in its absence for 96 h, and then cell numbers were counted. (**E**) qRT-PCR analysis of RNAPIII transcripts in WT cells incubated in the presence of under ML60218 or in its absence for 72 h. Transcripts levels were normalized to the levels in the absence of ML60218. Data represent means and individual data points (n = 2). (**F**) Survival of WT cells after cisplatin treatment. WT cells were incubated in the presence of BMH21 or in its absence for several days, and then, the indicated concentration of cispatin was added. Cell numbers were counted 72 h after the addition and normalized to the numbers of untreated cells. Data represent mean ± S.D. (n = 3).

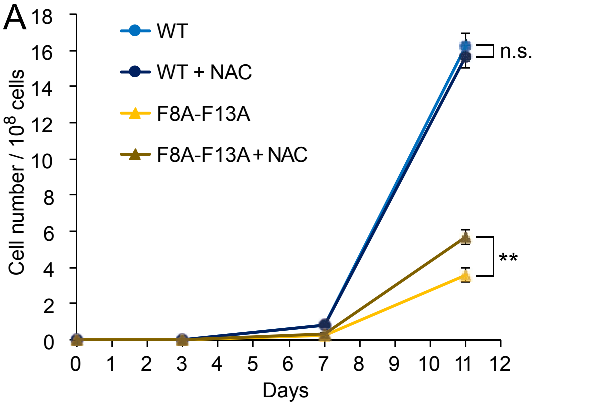

	WT	WT	F8A-F13A	F8A-F13A
3 µM NAC	–	+	–	+
Doubling time (h)	22.6	22.7	27.8	26.0

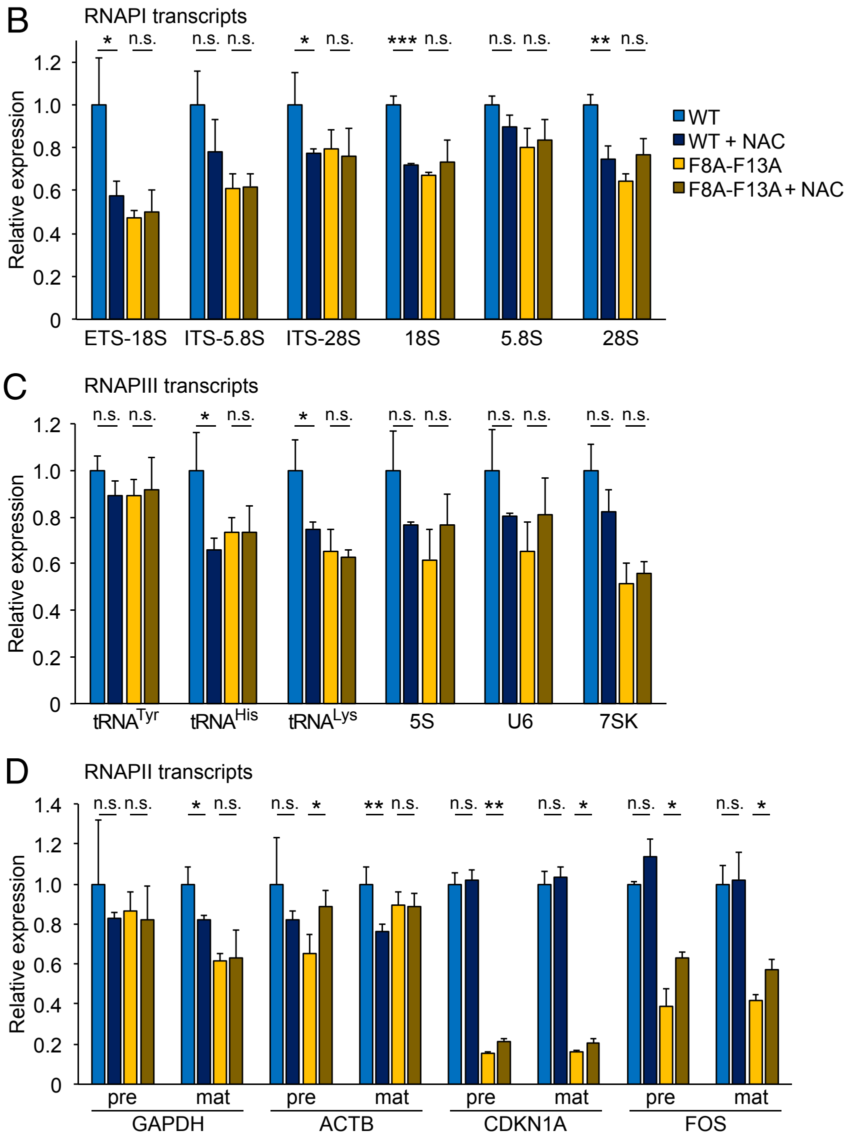

Supplementary Figure S14. NAC partially restores growth defects in HeLa cells expressing F8A-F13A. (**A**) Growth curves of HeLa cells expressing RPB6 WT or F8A-F13A in the presence of 3 µM NAC or in its absence. Cell numbers were counted at days 0, 3, 7, and 11. Doubling time was calculated from the growth curves and is indicated in the lower table. Data represent mean ± S.D. (n = 3). n.s., not significant; **, $p < 0.01$ (two-tailed Student's t-test). (**B**–**D**) qRT-PCR analysis of RNAPI (**B**), RNAPIII (**C**), and RNAPII (**D**) transcripts in HeLa cells expressing RPB6 WT or F8A-F13A that were incubated in the presence of 3 µM NAC or in its absence for several days. Transcript levels were normalized to the levels of WT without NAC. Pre and mat indicate precursor and mature transcripts, respectively. Data represent mean ± S.D. (n = 3). *, $p < 0.05$; **; $p < 0.01$; ***, $p < 0.001$ (two-tailed Student's t-test).

Supplementary Table S1. Oligonucleotide sequences used in this study.

**shRNAs against *RPB6***	
RPB6_sh-1s	5′-ACCGGGAAGCGAATCATCACACTATAGTTAATATTCATAGCTATGGTGTGGTGATTCGCTTCTTT-3′
RPB6_sh-1as	5′-GAAAAAAGAAGCGAATCACCACACCATAGCTATGAATATTAACTATAGTGTGATGATTCGCTTCC-3′
RPB6_sh-2s	5′-ACCGGGATGATTTGGAGAATGCTGAAGTTAATATTCATAGCTTCGGCATTCTCCAAGTCATCTTT-3′
RPB6_sh-2as	5′-GAAAAAAGATGACTTGGAGAATGCCGAAGCTATGAATATTAACTTCAGCATTCTCCAAATCATCC-3′
RPB6_sh-3s	5′-ACCGGGAAGGAACTTAAGGCCTGAAAGTTAATATTCATAGCTTTCGGGCCTTGAGTTCCTTCTTT-3′
RPB6_sh-3as	5′-GAAAAAAGAAGGAACTCAAGGCCCGAAAGCTATGAATATTAACTTTCAGGCCTTAAGTTCCTTCC-3′
**qRT-PCR analysis for *RPB6***	
RPB6_total_Fwd	5′-ATGACCAAGTACGAGCGAGC-3′
RPB6_total_Rev	5′-CTCCCATCTGGCAGGTAACG-3′
RPB6_endo_Fwd	5′-GCCAACCAGAAGCGAATCAC-3′
RPB6_endo_Rev	5′-CTTTCGGGCCTTGAGTTCCT-3′
RPB6_exo_Fwd	5′-CCAAAAACGGATTACAACCCCC-3′
RPB6_exo_Rev	5′-TCTTCCGTGCTTTCAGCTCT-3′
**qRT-PCR analysis for RNAPI-transcribed genes**	
ETS-18S_Fwd	5′-TCGCCGCGCTCTACCTTA-3′
ETS-18S_Rev	5′-CTGTACCGGCCGTGCGTA-3′
ITS1-5.8S_Fwd	5′-CTCGCCAAATCGACCTCGT-3′
ITS1-5.8S_Rev	5′-AAGTGTCGATGATCAATGTGTC-3′
ITS2-28S_Fwd	5′-GCGATTCCGTCCGTCCGT-3′
ITS2-28S_Rev	5′-CTGGTTAGTTTCTTCTCCTCCG-3′
5.8S_rRNA_Fwd	5′-ACTCTTAGCGGTGGATCACTCG-3′
5.8S_rRNA_Rev	5′-CGAAGTGTCGATGATCAATGTG-3′
18S_rRNA_Fwd	5′-ACGACCCATTCGAACGTCTG-3′
18S_rRNA_Rev	5′-TCTCCGGAATCGAACCCT-3′
28S_rRNA_Fwd	5′-GCCGAAACGATCTCAACCTA-3′
28S_rRNA_Rev	5′-CCTTTTCTGGGGTCTGATGA-3′
**qRT-PCR analysis for RNAPII-transcribed genes**	
pre_GAPDH_Fwd	5′-AGAGCTCAAGGTCAGCGCTC-3′
pre_GAPDH_Rev	5′-CTAGGGGAAGGAGGCTCC-3′
pre_ACTB_Fwd	5′-AATCTGGCACCACACCTTCTACA-3′
pre_ACTB_Fwd	5′-GGCAGAAGAGAGAACCAGTGAGA-3′
pre_p21_Fwd	5′-TGCCGAAGTCAGTTCCTTGT-3′
pre_p21_Rev	5′-TCTCACCTCCTCTGAGTGCC-3′
pre_c-fos_Fwd	5′-AACTTCATTCCCACGGTCACTGC-3′
pre_c-fos_Rev	5′-AGTGGCTTCATCCTCTGTACTG-3′
mat_GAPDH_Fwd	5′-CTGGCGTCTTCACCACCATGG-3′
mat_GAPDH_Rev	5′-CATCACGCCACAGTTTCCCGG-3′
mat_ACTB_Fwd	5′-GAGGTGATAGCATTGCTTTCG-3′
mat_ACTB_Rev	5′-CAAGTCAGTGTACAGGTAAGC-3′
mat_p21_Fwd	5′-AAGACCATGTGGACCTGTCAC-3′
mat_p21_Rev	5′-AGACTAAGGCAGAAGATGTAG-3′
mat_c-fos_Fwd	5′-CACTCCAAGCGGAGACAGAC-3′
mat_c-fos_Rev	5′-GAGCTGCCAGGATGAACTCT-3′
**qRT-PCR analysis for RNAPIII-transcribed genes**	
tRNA-Tyr-GTA_Fwd	5′-TGTAGATCCTTAGGTCGCTGG-3′
tRNA-Tyr-GTA_Rev	5′-TCCTTCGAGCCGGAATCG-3′
tRNA-His-GTG_Fwd	5′-TTGTGGCCGCAGCAACCT-3′
tRNA-His-GTG_Rev	5′-TGCCGTGACTCGGATTCG-3′
tRNA-Lys-UUU_Fwd	5′-GTCGGTAGAGCATCAGACTT-3′
tRNA-Lys-UUU_Rev	5′-CCTGGACCCTCAGATTAAAA-3′
7SK_Fwd	5′-ATTGATCGCCAGGGTTGATTC-3′
7SK_Rev	5′-AATGGACCTTGAGAGCTTGTTTG-3′
U6_Fwd	5′-CTCGCTTCGGCAGCACA-3′
U6_Rev	5′-AACGCTTCACGAATTTGCGT-3′
5S_rRNA_Fwd	5′-GTCTACGGCCATACCACCCTG-3′
5S_rRNA_Rev	5′-GCCTACAGCACCCGGTATTCC-3′
**qRT-PCR analysis for *Spodoptera frugiperda GAPDH***	
Insect_GAPDH_Fwd	5′-TGTTGACGGACCCTCTGGAA-3′
Insect_GAPDH_Rev	5′-ACGGGAACACGGAAAGCCAT-3′
